# Exploring van der Waals materials with high anisotropy: geometrical and optical approaches

**DOI:** 10.1038/s41377-024-01407-3

**Published:** 2024-03-08

**Authors:** Aleksandr S. Slavich, Georgy A. Ermolaev, Mikhail K. Tatmyshevskiy, Adilet N. Toksumakov, Olga G. Matveeva, Dmitriy V. Grudinin, Kirill V. Voronin, Arslan Mazitov, Konstantin V. Kravtsov, Alexander V. Syuy, Dmitry M. Tsymbarenko, Mikhail S. Mironov, Sergey M. Novikov, Ivan Kruglov, Davit A. Ghazaryan, Andrey A. Vyshnevyy, Aleksey V. Arsenin, Valentyn S. Volkov, Kostya S. Novoselov

**Affiliations:** 1 Moscow Center for Advanced Studies, Kulakova str. 20, Moscow, 123592 Russia; 2 Emerging Technologies Research Center, XPANCEO, Internet City, Emmay Tower, Dubai, United Arab Emirates; 3 Donostia International Physics Center (DIPC), Donostia/San-Sebastián, 20018 Spain; 4 Institute of Materials, École Polytechnique Fédérale de Lausanne, 1015 Lausanne, Switzerland; 5 Department of Chemistry, Lomonosov Moscow State University, Moscow, 119991 Russia; 6 Laboratory of Advanced Functional Materials, Yerevan State University, Yerevan, 0025 Armenia; 7 National Graphene Institute (NGI), University of Manchester, Manchester, M13 9PL UK; 8 Department of Materials Science and Engineering, National University of Singapore, Singapore, 03-09 EA Singapore; 9 Institute for Functional Intelligent Materials, National University of Singapore, 117544 Singapore, Singapore

**Keywords:** Optical properties and devices, Silicon photonics

## Abstract

The emergence of van der Waals (vdW) materials resulted in the discovery of their high optical, mechanical, and electronic anisotropic properties, immediately enabling countless novel phenomena and applications. Such success inspired an intensive search for the highest possible anisotropic properties among vdW materials. Furthermore, the identification of the most promising among the huge family of vdW materials is a challenging quest requiring innovative approaches. Here, we suggest an easy-to-use method for such a survey based on the crystallographic geometrical perspective of vdW materials followed by their optical characterization. Using our approach, we found As_2_S_3_ as a highly anisotropic vdW material. It demonstrates high in-plane optical anisotropy that is ~20% larger than for rutile and over two times as large as calcite, high refractive index, and transparency in the visible range, overcoming the century-long record set by rutile. Given these benefits, As_2_S_3_ opens a pathway towards next-generation nanophotonics as demonstrated by an ultrathin true zero-order quarter-wave plate that combines classical and the Fabry–Pérot optical phase accumulations. Hence, our approach provides an effective and easy-to-use method to find vdW materials with the utmost anisotropic properties.

## Abstract (web summary)

We developed a geometric approach to identify highly anisotropic materials. This leads to the discovery of giant anisotropy of As_2_S_3_, enabling the record-small quarter-wave plate.

## Graphical abstract

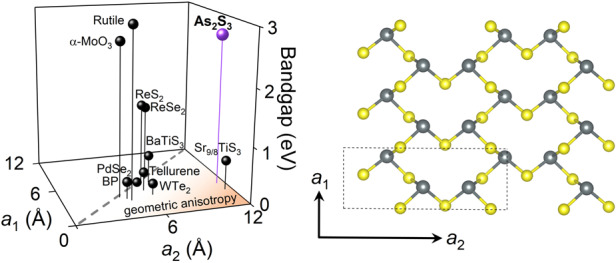

## Introduction

Modern nanophotonics exploits a plethora of novel phenomena for advanced light manipulation. Among them are bound states in the continuum^1^, chirality-preserving reflection^2^, virtual-reality imaging^3,4^, and others. The key parameter in these effects is the refractive index *n* since it governs the resonance wavelength *λ*_res_ (${\lambda }_{{\rm{res}}} \sim 1/n$)^5^ and the resonance quality factor *Q* (e.g., *Q ~ n*^2^ for the Mie resonances)^6^ and the optical power, which is proportional to *n* − 1. Hence, even a slight increase in the refractive index gives a tremendous advantage in optical applications^7^. However, the refractive index is fundamentally limited^7^, with the best results provided by high-refractive index materials, such as Si^8^, GaP^9^, TiO_2_^10^, InGaS_3_^11^, and SnS_2_^12^. Nevertheless, a certain component or components of a material’s refractive index can be further increased by sacrificing the other component^7^, resulting in a novel family of materials which possess the highest refractive indices and highest anisotropy at the same time. Although optical anisotropy has been known for a long time, in recent years, novel applications enabled particularly by high anisotropy have emerged^13–19^. Optical anisotropy revolutionizes integrated nanophotonics^20^ by enabling subdiffractional light guiding^21–25^, polariton canalization^26,27^, Dyakonov surface waves^28^, and the high integration density of waveguides^29,30^.

The most promising anisotropic materials are van der Waals (vdW) crystals^15,31–33^. They are bulk counterparts of two-dimensional (2D) materials. Their 2D layered origin naturally leads to record values of optical anisotropy^15^ because of the fundamental difference between in-plane covalent and out-of-plane vdW atomic bonds. However, it mostly results in out-of-plane birefringence, while some interesting effects require in-plane anisotropy^16,34^. At present, rutile continues to hold the record for strongest in-plane optical anisotropy in the visible range despite the significant advances in materials science^16–18,35^, which is surprising considering that several decades have passed since its discovery^36^. It has inspired intensive research of low-symmetry vdW crystals^37–42^.

In this work, we provide a solution to this long-term challenge. Our consideration of lattice geometry reveals that arsenic trisulfide (As_2_S_3_) stands out among other low-symmetry crystals. Further study of this material by micro-transmittance spectroscopy and spectroscopic ellipsometry in combination with quantum-mechanical computations confirmed that it possesses a high in-plane optical anisotropy in the visible range. They also show that As_2_S_3_ belongs to transparent high-refractive index materials. Thus, As_2_S_3_ offers a universal material platform for nanooptics that brings benefits of both high optical anisotropy and high refractive index.

## Results

### Origins of high optical anisotropy of van der Waals materials

Recent investigations^32,43^ of vdW materials’ optical properties reveal that those constitute the next-generation high refractive index materials platform with about 80% larger polarizability compared to traditional photonic materials, such as Si, GaP, and TiO_2_. Hence, the search for highly refractive materials in the visible range among vdW crystals is a natural next step. Nevertheless, it is a tedious task because there are more than 5000 vdW crystals^44^, and a straightforward enumeration of options is unacceptably time-consuming. To reduce the search area and pick the most promising materials for nanophotonics, we particularly aim for high in-plane optical anisotropy. Still, the family of anisotropic vdW crystals is huge, which motivates us to identify features relevant to large optical anisotropy. This problem is challenging since optical anisotropy could result from numerous unrelated physical effects. Among them are preferential directions of excitons^15,18,45^, atomic-scale modulations^17^, quasi-one-dimensional structures^16,46^, different natures of atomic bonding^15^, aligned interaction of dipole excitations around specific atoms^47^, phonon resonances^14,42,48,49^, and many others. Obviously, one of the reasons for the strong optical anisotropy is the directional material resonances, such as excitons^15,18,45^ and phonons^14,48^. However, it is difficult to identify directional resonances or types of atomic bonding since it requires costly quantum-mechanical simulations. Therefore, we choose an alternative approach, and assume that excitations with very strong anisotropy can manifest themselves in or be caused by the geometrical anisotropy of an elementary crystal cell.

**Fig. 1:**
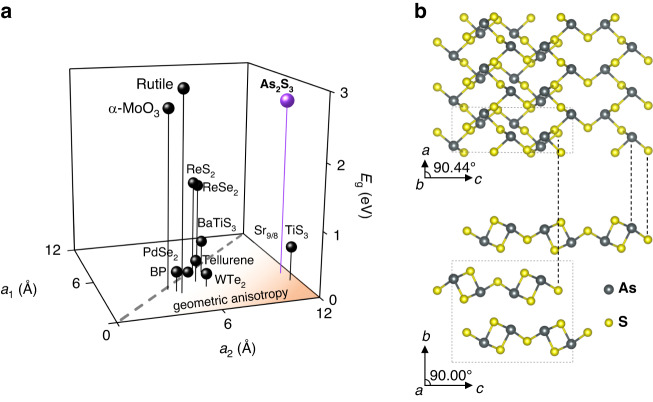
Anisotropic crystalline structure as an origin for high optical anisotropy. **a** The comparison of the lattice parameters of the representative anisotropic crystals and their bandgaps. *a*_1_ and *a*_2_ stand for the in-plane lattice parameters. **b** Monoclinic crystal structure of As_2_S_3_, viewed along the crystallographic *b*-axis (top) and *a*-axis (bottom). The black dashed frame shows the unit cell

Next, we compare the crystal structure of the most representative in-plane anisotropic crystals in Fig. 1a. Interestingly, two materials stand out, namely, Sr_9/8_TiS_3_ and As_2_S_3_. According to Fig. 1a, they exhibit the largest “geometric anisotropy”, that is, the ratio of in-plane lattice parameters. Indeed, a recent study^17^ shows that Sr_9/8_TiS_3_ has the largest optical anisotropy (Δ*n* ~ 2.1) with zero losses, which coincides with our predictions. However, the optical bandgap of Sr_9/8_TiS_3_ corresponds to the infrared spectral range, which makes it less suitable for visible range applications. In contrast, the optical bandgap of As_2_S_3_ is within the visible range with *E*_g_ ~2.7 eV (*λ*_g_ ~460 nm)^50,51^, and As_2_S_3_ crystal (Fig. 1b) demonstrates a similar to Sr_9/8_TiS_3_ “geometric anisotropy”. Consequently, we anticipate that As_2_S_3_ will offer a high optical anisotropy and, like other vdW materials with strong optical anisotropy^43^, a high refractive index in the visible range.

### Crystal structure of van der Waals As_2_S_3_

As_2_S_3_ is a yellow semiconducting crystal usually found in nature as the mineral orpiment^50^. Amorphous As_2_S_3_ has already proved useful in such photonic applications as holography^52^ and fibers^53^. At the same time, As_2_S_3_ in vdW configuration (see Fig. 1b and Supplementary Note 1 for As_2_S_3_ characterization) appeared only recently in the research focus owing to the extraordinarily large in-plane mechanical anisotropy^54,55^. It also shows that our approach based on lattice geometry consideration in Fig. 1a applies to other anisotropic properties beyond the optical one.

**Fig. 2:**
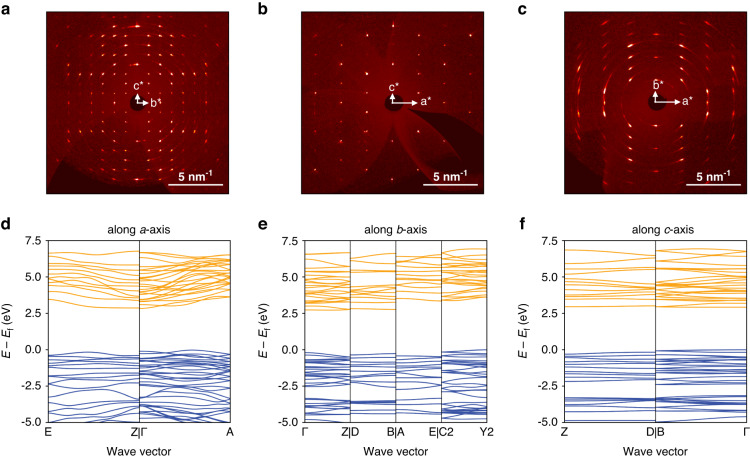
As_2_S_3_ anisotropic crystal structure, a comprehensive characterization. XRD patterns in reciprocal space along **a** c*-b*, **b** c*-a*, and **c** b*-a* reciprocal planes. The electronic bandstructure cuts along **d** *a*-axis, **e** *b*-axis, and **f** *c*-axis. Orange and blue curves present conduction and valence bands, respectively

In light of the importance of lattice parameters, we commenced our study of As_2_S_3_ with their refinement *via* X-ray diffraction measurements (see Methods). The XRD imaging patterns in Fig. 2a–c confirm the monoclinic structure of As_2_S_3_ (see Fig. 1b) with the following lattice parameters: $a=4.2546(4)$ Å, $b=9.5775(10)$ Å, $c=11.4148(10)$ Å, $\alpha =90$°, $\beta =90.442(4)$°, and $\gamma =90$°. Using these values of the parameters, we computed the bandstructure of As_2_S_3_ (see Supplementary Note 2) from the first principles (see Methods). Of great interest are the bandstructure cuts along crystallographic axes, presented in Fig. 2d–f. First of all, we notice a considerable difference in the dispersion curves, which clearly indicates significant anisotropic properties. Moreover, the bandstructure cuts for in-plane directions have a fundamental difference: along the *a*-axis, the bandstructure has a high dispersion, while it is flat along the *c*-axis. Therefore, we expect a stronger dielectric response for the *c*-axis than for the *a*-axis as flat bands lead to a large density of states and, as a result, a large refractive index^7,56^.

### In-plane optical anisotropy of van der Waals As_2_S_3_

**Fig. 3:**
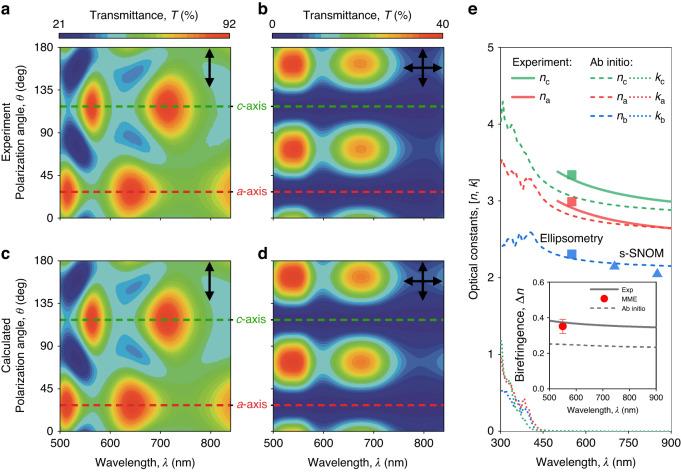
Optical properties of van der Waals As_2_S_3_. Experimental polarized micro-transmittance spectra of an As_2_S_3_ flake for **a** parallel- and **b** cross-polarized configurations. Calculated polarized micro-transmittance of As_2_S_3_ for **c** parallel- and **d** cross-polarized configurations. The dashed lines show the position of crystallographic axes *a* (red line) and *c* (green line). **e** Anisotropic optical constants of As_2_S_3_. The inset shows the in-plane birefringence of As_2_S_3_. Tabulated optical constants of As_2_S_3_ are collected in Supplementary Note 7

In general, for monoclinic systems, anisotropic permittivity tensor has a diagonal form diag(*n*_*a*_, *n*_*b*_, *n*_*c*_) in the crystallographic (*a*, *b*, *c*) basis, where *n*_*a*_, *n*_*b*_, and *n*_*c*_ are refractive indices along the corresponding crystallographic axes^57^. The problem with this description is a non-orthogonal (*a*, *b*, *c*) basis, which significantly complicates the determination and the use of monoclinic optical constants since it is impossible to decouple the contribution of *n*_*a*_, *n*_*b*_, and *n*_*c*_ into the optical response of the monoclinic crystal. Luckily, the monoclinic angle *β* of As_2_S_3_ differs from 90° very slightly by just 0.442(4)°, which allows us to treat As_2_S_3_ as an orthorhombic crystal. In this approximation, we can separately probe As_2_S_3_ optical components (*n*_*a*_, *n*_*b*_, and *n*_*c*_) by orthogonal polarizations. For this purpose, we measured the polarized micro-transmittance of As_2_S_3_ flakes exfoliated on Schott glass substrates (see Methods) and determined their crystallographic axes by polarized Raman spectroscopy (see Supplementary Note 3). The exemplified transmittance spectra maps of 345-nm-thick flake for parallel- and cross- polarizations are plotted in Fig. 3a, b. Note that we choose the transparency range (500 – 850 nm) of As_2_S_3_, which allows us to leverage the Cauchy models for As_2_S_3_ refractive indices^15^. Using this description, we fitted the experimental data (see Fig. 3a, b), and calculated the corresponding spectra in Fig. 3c, d. Calculations agree perfectly with the experiment (Fig. 3a, b) and give us in-plane optical constants of As_2_S_3_ presented in Fig. 3e. However, micro-transmittance spectroscopy cannot probe the out-of-plane component of the As_2_S_3_ dielectric tensor. Therefore, we performed single-wavelength Mueller-Matrix ellipsometry and near-field studies (see Supplementary Notes 4–6) presented in Fig. 3e to get the complete picture of the As_2_S_3_ dielectric response. Additionally, we performed the first-principle calculations (see Methods) of the As_2_S_3_ dielectric function (see Fig. 3e), which coincides well with the measured values, especially for in-plane components, *n*_*a*_ and *n*_*b*_. Notably, even first-principle computations yield zero extinction coefficient *k* for the considered visible range (see Fig. 3e), which confirms that As_2_S_3_ is a lossless material, promising for visible nanophotonics.

### As_2_S_3_ in the family of high-refractive index materials

**Fig. 4:**
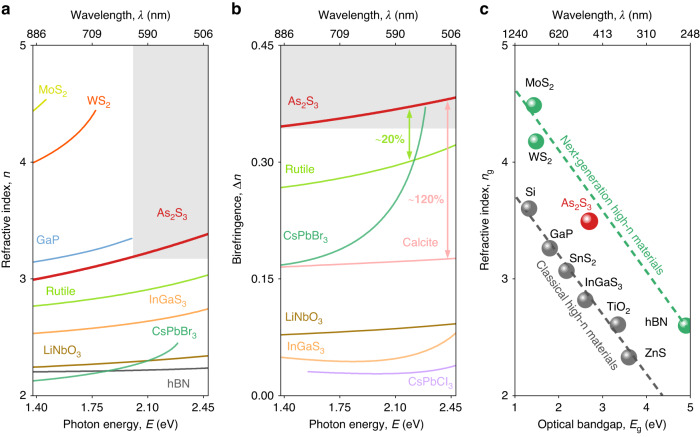
As_2_S_3_ in a family of high refractive index and birefringent materials. **a** Refractive index and **b** the birefringence of van der Waals As_2_S_3_ and conventional photonic materials in their transparency windows. **c** Comparison of the maximum in-plane refractive index of van der Waals As_2_S_3_ in the transparency window with the established highly refractive materials

Of immediate interest are absolute values of As_2_S_3_ optical constants (see Figs. 3e and 4a, b). As anticipated from the bandstructure calculations in Fig. 2d–f, As_2_S_3_ has the largest refractive index *n*_*c*_ along the crystallographic *c*-axis (see Fig. 3e). Besides, the benchmarking of *n*_*c*_ with other crystals (Fig. 4a) reveals that As_2_S_3_ also belongs to the family of high refractive index materials and holds record values below 620 nm. Extrapolating the Cauchy model for *n*_*c*_ to As_2_S_3_ optical bandgap (*E*_g_ ~2.7 eV), we can clearly see that As_2_S_3_ fits the correlation between the optical bandgap and refractive index for vdW materials, as shown in Fig. 4c.

Apart from a high refractive index, As_2_S_3_ possesses high in-plane optical anisotropy *Δn* ~0.4 (see the inset in Fig. 3e). This is 20% greater than the birefringence of rutile and even outperforms the excitonic maximum anisotropy of CsPbBr_3_ perovskite^18^, as seen in Fig. 4b. More importantly, the birefringence of As_2_S_3_ is over double of the calcite’s one—a traditional anisotropic crystal (Fig. 4b). Therefore, Fig. 4 demonstrates that As_2_S_3_ combines both high refractive index and high optical anisotropy with zero optical losses up to material’s bandgap of 2.7 eV (460 nm), which are the most crucial factors in the state-of-the-art nanophotonics thanks to the high geometrical anisotropy.

Still, the correlation between the geometric anisotropy and the optical anisotropy is not that straightforward. This is because geometrical anisotropy is an essential and important factor, but not the only one that determines optical anisotropy. For example, the bandgap also strongly influences the optical anisotropy, which is the primary reason for including the bandgap into consideration with geometrical parameters of crystals in Fig. 1a. As seen in Fig. 4c, the larger the bandgap, the smaller the refractive index. Similarly, since optical anisotropy is, by definition, the difference between refractive indices along corresponding directions, the relation between the bandgap and optical anisotropy remains the same: the larger the bandgap, the smaller the optical anisotropy. This fact explains why similar geometric anisotropies of As_2_S_3_ and Sr_9/8_TiS_3_ result in different optical anisotropy values. Furthermore, it also makes clear why rutile and calcite held the record for the highest optical anisotropy for several decades.

### Unconventional true zero-order quarter-wave plates based on van der Waals As_2_S_3_

**Fig. 5:**
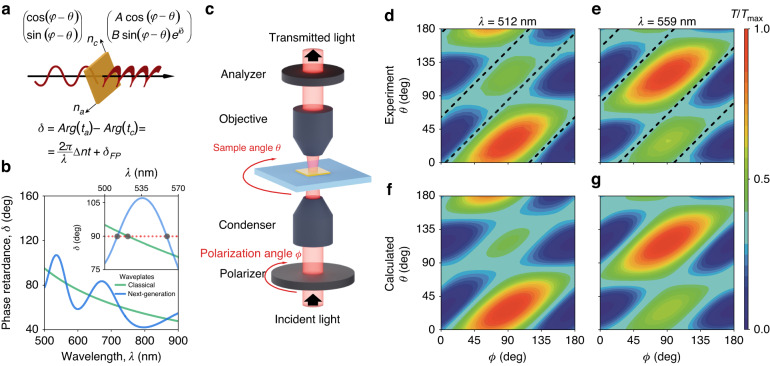
True zero-order quarter-wave plates based on ultrathin van der Waals As_2_S_3_. **a** The concept of As_2_S_3_ wave plate: a combination of “classical” phase accumulation and Fabry–Pérot contribution arising from high optical anisotropy. **b** The comparison of phase retardance between classical and As_2_S_3_-based true zero-order wave plates. **c** Schematic representation of the experimental setup. Measured polarized transmittance countourplot at **d** 512 nm and **e** 559 nm. The dashed lines show the quarter-wave plate operation regime. The data are normalized to the maximum values for each wavelength of transmitted light throughout the figure. Transmittance calculations are based on the anisotropic dielectric function presented in Fig. 3e at **f** 512 nm and **g** 559 nm

The exceptional optical properties of As_2_S_3_ (see Fig. 4) not only make this crystal promising for next-generation nanophotonics but also change the operation principle of classical optical elements. Besides, As_2_S_3_ wave plates do not require specific offcut at angles off normal from the crystal axes, unlike most wave plate materials, including calcite. This fact simplifies the design and fabrication since the modification of operational wavelength for As_2_S_3_ wave plates only requires a change in their thickness. To demonstrate this, we investigated the wave plate characteristics of the As_2_S_3_ flake. Traditionally, the retardance *δ* between the fast- and slow-axes of an anisotropic wave plate is determined by the simple expression 2*π∆nt/λ*, where *λ* is the wavelength of light in vacuum, ∆*n* defines the material’s birefringence, and *t* denotes the wave plate’s thickness. However, this formula only holds for small values of *∆n* since it disregards the light scattering at the faces of an anisotropic material. Due to the significant difference in the refractive index components along the principal directions of the wave plate, the phase accumulation due to the repeated Fabry–Pérot reflections makes the full retardance deviate from the simplified formula (see Fig. 5a and Supplementary Notes 8, 9). As a result, the high optical anisotropy enables quarter-wave retardance at multiple wavelengths and at a thickness that is lower than predicted by the simplified expression. For instance, our As_2_S_3_ operates as a true zero-order quarter-wave plate at two wavelengths (512 and 559 nm), and at 559 nm, its thickness is lower than expected from the simplified expression. In contrast, the simplified equation predicts only single-wavelength operation at 522 nm (see Fig. 5b). Here, we utilized a micro-transmittance scheme (see Fig. 1c) at 512 and 559 nm to check this concept. The resulting transmittance maps in Fig. 5d–g confirm the predicted quarter-wave plate behavior for the selected wavelengths. Furthermore, Fig. 5d–g demonstrates that the polarization angles responsible for a quarter-wave plate mode differ from 45° with respect to the principal optical axes, unlike classical quarter-wave plates with 45° orientation^34^. This effect also originates from the high optical anisotropy, which results in unequal absolute values of transmission amplitudes, *A* and *B*, for the light polarized along principal directions (see Fig. 5a). It explains our observations from Fig. 5d–g. Finally, we would like to note that our quarter-wave plate has an extremely small thickness of 345 nm compared to the previous record-holder of ferrocene-based true zero-order quarter-wave plate with 1071 nm thickness operating at 636 nm wavelength^34^. Hence, our device has about threefold improvement in size, which brings us a step closer to miniaturized next-generation optical elements.

## Discussion

In summary, we provide a new route for exploring anisotropic vdW materials by comparing their crystal structures and bandgaps. In combination with optical characterization, it becomes a convenient tool for a quick assessment of promising vdW materials for anisotropy-based applications^13–16,18,19^. Our approach reveals that As_2_S_3_ is a perfect vdW material for visible range nanophotonics with the largest in-plane optical anisotropy, high refractive index, and zero optical losses down to 460 nm. These properties enrich photonic applications with a variety of novel possibilities at the nanoscale. In particular, the high optical anisotropy of As_2_S_3_ could be used for the realization of chiral optical response in planar structures^58^, nontrivial topological phase singularities^59^, extreme skin depth waveguiding^21,22^, reduction of crosstalk between waveguides^43^, and design simplification of metasurfaces or metamaterials with anisotropic optical response^60^. For example, we designed an ultrathin two-wavelength As_2_S_3_-based quarter-wave plate, which is three times more compact than the thinnest single-wavelength quarter-wave plate^34^. Furthermore, our anisotropy analysis can be used beyond vdW materials. For instance, our analysis predicts large anisotropy for non-vdW Sr_9/8_TiS_3_, which was recently discovered to have colossal optical anisotropy in the near-infrared range^17^. Moreover, we expect large optical anisotropy in other crystals with high geometrical anisotropy, such as As_2_Se_3_ (*a*_1_/*a*_2_ ≈ 2.8) for visible and near-infrared wavelengths, Ta_2_NiSe_5_ (*a*_1_/*a*_2_ ≈ 4.5) and Ta_2_NiSe_5_ (*a*_1_/*a*_2_ ≈ 4.4)^35^ for infrared wavelengths. Besides, anisotropy of mechanical, electronic, optical, and other properties are closely connected, which allows the application of the proposed method for other topics. Indeed, As_2_S_3_ also demonstrates high mechanical anisotropy^54,55^ in addition to the high optical anisotropy revealed in our work. Therefore, our findings can lead to the rapid development of low-symmetry materials^37–40^ by establishing a milestone for their anisotropy evaluation.

## Materials and methods

### Sample preparation

Bulk synthetic As_2_S_3_ crystals were purchased from 2d semiconductors (Scottsdale) and exfoliated on top of Si, Si/SiO_2_, quartz, and Schott glass substrates at room temperature by commercial scotch tapes from Nitto Denko Corporation (Osaka, Japan). Prior to exfoliation, the corresponding substrates were subsequently cleaned in acetone, isopropanol alcohol, and deionized water, and then, subjected to oxygen plasma (O_2_) to remove the ambient adsorbates.

### Atomic-force microscopy characterization

The thickness of As_2_S_3_ flakes was accurately characterized by an atomic-force microscope (NT-MDT Ntegra II) operated in contact mode at ambient conditions. AFM measurements were acquired using silicon tips (ETALON, HA_NC ScanSens) with a head curvature radius of <10 nm, a spring constant of 3.5 N m^−1^, and a resonant frequency of 140 kHz. Gwyddion software was used for image processing and quantitative analysis.

### X-ray diffraction analysis

X-ray diffraction analysis of As_2_S_3_ single crystal was performed on a Bruker D8 QUEST diffractometer with a Photon III CMOS area detector using Mo Kα radiation (*λ* = 0.71073 Å) focused by a multilayer Montel mirror. Full data set was collected at 293 K within *φ*- and *ω*-scans applying sample-to-detector distance of 80 and 100 mm to improve the precision of refined unit cell parameters. Raw data were indexed with cell_now and integrated using SAINT from the SHELXTL PLUS package^61,62^. Absorption correction was performed using a numerical method based on crystal shape as implemented in SADABS. Crystal structure was solved by direct methods and refined anisotropically with the full-matrix F2 least-squares technique using SHELXTL PLUS. Further details of the data collection and refinement parameters are summarized in Table S1. Selected interatomic distances and bond angles are listed in Table S2. It is worth noting that the crystal structure of monoclinic As_2_S_3_ was previously reported^63,64^ in a non-conventional unit cell setting, which can be transformed to the conventional setting by $$\left(\begin{array}{ccc}0 & 0 & 1\\ 0 & -1 & 0\\ 1 & 0 & 0\end{array}\right)$$ matrix. Unit cell parameters and atomic positions reported in the present work were determined with higher precision (Table S3). CSD reference number 2258216 contains supplementary crystallographic data for this paper. These data can be obtained free of charge from the Cambridge Crystallographic Data Centre via www.ccdc.cam.ac.uk/data_request/cif.

### First-principle calculations

Electronic bandstructure calculations were performed using the screened hybrid functional HSE06 with 25% of mixing as implemented in Vienna ab initio simulation package (VASP) code^65,66^. The core electrons are described with projector augmented wave (PAW) pseudopotentials treating the As 4*s* and 4*p* and the S 3*s* and 3*p* electrons as valence. A kinetic energy cutoff for the plane-wave basis was set to 350 eV. To calculate bandstructure we generated a path in reciprocal space using Spglib and SeeK-path and used a standardized primitive cell by conventions of Spglib. Optical properties of As_2_S_3_ were calculated using HSE06 hybrid functional. For this, we used Г-centered k-points mesh sampling the Brillouin zone with a resolution of 2π ∙ 0.05 Å^−1^. Optical properties were calculated within GW approximation on wavefunctions calculated using HSE06 hybrid functional using the VASP code. For this, we obtained ground-state one-electron wavefunctions from HSE06 and used them to start the GW routines. Finally, we calculated the imaginary and real parts of the frequency-dependent dielectric function within the GW approximation.

### Angle-resolved micro-transmittance

The spectroscopic transmittance was measured in the 500–900 nm spectral range on an optical upright microscope (Zeiss Axio Lab.A1) equipped with a halogen light source, analyzer, polarizer, and grating spectrometer (Ocean Optics QE65000) coupled by optical fiber (Thorlabs M92L02) with core diameter 200 µm. The transmitted light was collected from a spot of <15 µm using an objective with ×50 magnification and numerical aperture N.A. = 0.8 (Objective “N-Achroplan” 50×/0.8 Pol M27). A detailed description of the micro-transmittance setup can be found in publication^67^.

### Imaging Mueller-matrix ellipsometry

A commercial Accurion nanofilm_ep4 ellipsometer (Accurion GmbH) was used to measure 11 elements of the Mueller-matrix (*m*_12_, *m*_13_, *m*_14_, *m*_21_, *m*_22_, *m*_23_, *m*_24_, *m*_31_, *m*_32_, *m*_33_, *m*_34_). The measurements were carried with a 5° sample rotation angle step, 550 nm incident light wavelength, and 50° incident angle in rotation compensator mode.

### Scanning near-field optical microscopy

Near-field imaging was performed using a commercially available scattering-type Scanning Near Field Optical Microscope (neaSNOM), which allows to simultaneously scan the topography of the sample along with the amplitude and phase of the near-field signal. For the illumination of the sample, we used a tunable Ti:Sapphire laser (Avesta) with a wavelength in the spectral range of 700–1000 nm. The measurements were conducted using reflection mode. As a scattering probe, we used a platinum/iridium5 (PtIr_5_) coated AFM tip (ARROW-NCPt-50, Nanoworld) with a resonant frequency of about 275 kHz and a tapping amplitude of 100 nm.

### Supplementary information

Supplementary Information

## Data Availability

The relevant raw and generated data supporting the key findings of this study are available in the figshare database under accession code https://figshare.com/s/57877dc20f994e7cd8c1 (10.6084/m9.figshare.24967677). All data are available from the corresponding author upon request.

## References

[1] Koshelev K (2018). Asymmetric metasurfaces with high-*Q* resonances governed by bound states in the continuum. Phys. Rev. Lett..

[2] Voronin K (2022). Single-handedness chiral optical cavities. ACS Photonics.

[3] Li ZY (2021). Meta-optics achieves RGB-achromatic focusing for virtual reality. Sci. Adv..

[4] Xiong JH (2021). Augmented reality and virtual reality displays: emerging technologies and future perspectives. Light Sci. Appl..

[5] Evlyukhin AB (2012). Demonstration of magnetic dipole resonances of dielectric nanospheres in the visible region. Nano Lett..

[6] Kruk S, Kivshar Y (2017). Functional meta-optics and nanophotonics governed by mie resonances. ACS Photonics.

[7] Khurgin JB (2022). Expanding the photonic palette: exploring high index materials. ACS Photonics.

[8] Staude I, Schilling J (2017). Metamaterial-inspired silicon nanophotonics. Nat. Photonics.

[9] Kuznetsov A (2023). Elastic gallium phosphide nanowire optical waveguides—versatile subwavelength platform for integrated photonics. Small.

[10] Sun S (2017). All-dielectric full-color printing with TiO_2_ metasurfaces. ACS Nano.

[11] Toksumakov AN (2022). High-refractive index and mechanically cleavable non-van der Waals InGaS_3_. npj 2D Mater. Appl..

[12] Ermolaev GA (2021). Broadband optical constants and nonlinear properties of SnS_2_ and SnSe_2_. Nanomaterials.

[13] Jahani S, Jacob Z (2016). All-dielectric metamaterials. Nat. Nanotechnol..

[14] Ma WL (2018). In-plane anisotropic and ultra-low-loss polaritons in a natural van der Waals crystal. Nature.

[15] Ermolaev GA (2021). Giant optical anisotropy in transition metal dichalcogenides for next-generation photonics. Nat. Commun..

[16] Niu SY (2018). Giant optical anisotropy in a quasi-one-dimensional crystal. Nat. Photonics.

[17] Mei HY (2023). Colossal optical anisotropy from atomic-scale modulations. Adv. Mater..

[18] Ermolaev G (2023). Giant and tunable excitonic optical anisotropy in single-crystal halide perovskites. Nano Lett..

[19] Krasnok A, Alù A (2022). Low-symmetry nanophotonics. ACS Photonics.

[20] Ling HN, Li RJ, Davoyan AR (2021). All van der Waals integrated nanophotonics with bulk transition metal dichalcogenides. ACS Photonics.

[21] Jahani S (2018). Controlling evanescent waves using silicon photonic all-dielectric metamaterials for dense integration. Nat. Commun..

[22] Ermolaev G (2022). Van der Waals materials for subdiffractional light guidance. Photonics.

[23] Ma WL (2021). Ghost hyperbolic surface polaritons in bulk anisotropic crystals. Nature.

[24] Hu CX (2023). Source-configured symmetry-broken hyperbolic polaritons. eLight.

[25] Zhang X (2023). Ultrafast anisotropic dynamics of hyperbolic nanolight pulse propagation. Sci. Adv..

[26] Hu GW (2020). Topological polaritons and photonic magic angles in twisted α-MoO_3_ bilayers. Nature.

[27] Duan JH (2020). Twisted nano-optics: manipulating light at the nanoscale with twisted phonon polaritonic slabs. Nano Lett..

[28] Takayama O (2009). Observation of dyakonov surface waves. Phys. Rev. Lett..

[29] Mia MB (2020). Exceptional coupling in photonic anisotropic metamaterials for extremely low waveguide crosstalk. Optica.

[30] Kabir MF (2023). Anisotropic leaky-like perturbation with subwavelength gratings enables zero crosstalk. Light Sci. Appl..

[31] Munkhbat B (2022). Nanostructured transition metal dichalcogenide multilayers for advanced nanophotonics. Laser Photonics Rev..

[32] Zotev PG (2023). Van der Waals materials for applications in nanophotonics. Laser Photonics Rev..

[33] Popkova AA (2022). Nonlinear exciton‐mie coupling in transition metal dichalcogenide nanoresonators. Laser Photonics Rev..

[34] Li ZP (2023). As‐grown miniaturized true zero-order waveplates based on low-dimensional ferrocene crystals. Adv. Mater..

[35] Feng YZ (2023). Visible to mid-infrared giant in-plane optical anisotropy in ternary van der Waals crystals. Nat. Commun..

[36] Glassford KM, Chelikowsky JR (1992). Optical properties of titanium dioxide in the rutile structure. Phys. Rev. B.

[37] Barraza-Lopez S (2020). Beyond graphene: low-symmetry and anisotropic 2D materials. J. Appl. Phys..

[38] Tian H (2016). Low-symmetry two-dimensional materials for electronic and photonic applications. Nano Today.

[39] Li L (2019). Emerging in‐plane anisotropic two‐dimensional materials. InfoMat.

[40] He MZ (2022). Anisotropy and modal hybridization in infrared nanophotonics using low-symmetry materials. ACS Photonics.

[41] Passler NC (2022). Hyperbolic shear polaritons in low-symmetry crystals. Nature.

[42] Hu GW (2023). Real-space nanoimaging of hyperbolic shear polaritons in a monoclinic crystal. Nat. Nanotechnol..

[43] Vyshnevyy AA (2023). Van der Waals materials for overcoming fundamental limitations in photonic integrated circuitry. Nano Lett..

[44] Mounet N (2018). Two-dimensional materials from high-throughput computational exfoliation of experimentally known compounds. Nat. Nanotechnol..

[45] Aslan B (2016). Linearly polarized excitons in single-and few-layer ReS_2_ crystals. ACS Photonics.

[46] Zhao BY (2022). Orientation-controlled anisotropy in single crystals of quasi-1D BaTiS_3_. Chem. Mater..

[47] Isherwood BJ, James JA (1976). Structural dependence of the optical birefringence of crystals with calcite and aragonite type structures. Acta Crystallogr. Sect. A.

[48] Dai S (2014). Tunable phonon polaritons in atomically thin van der Waals crystals of boron nitride. Science.

[49] Taboada-Gutiérrez J (2020). Broad spectral tuning of ultra-low-loss polaritons in a van der Waals crystal by intercalation. Nat. Mater..

[50] Zallen R, Slade ML, Ward AT (1971). Lattice vibrations and interlayer interactions in crystalline As_2_S_3_ and As_2_Se_3_. Phys. Rev. B.

[51] Ho CH (2011). Electronic structure and optical property of As_2_(Te_1−*x*_S_*x*_)_3_ and As_2_(Te_1−*x*_Se_*x*_)_3_ crystals. J. Alloy. Compd..

[52] Ozols A, Saharovs D, Reinfelde M (2006). Holographic recording in amorphous As_2_S_3_ films at 633nm. J. Non-Cryst. Solids.

[53] Gao WQ (2011). Visible light generation and its influence on supercontinuum in chalcogenide As_2_S_3_ microstructured optical fiber. Appl. Phys. Express.

[54] Šiškins M (2019). Highly anisotropic mechanical and optical properties of 2D layered As_2_S_3_ membranes. ACS Nano.

[55] Liu XF (2021). Highly anisotropic electronic and mechanical properties of monolayer and bilayer As_2_S_3_. Appl. Surf. Sci..

[56] Yu, P. Y. & Cardona, M. *Fundamentals of Semiconductors: Physics and Materials Properties* (Springer, 2010).

[57] Dressel M (2008). Kramers-kronig-consistent optical functions of anisotropic crystals: generalized spectroscopic ellipsometry on pentacene. Opt. Express.

[58] Voronin, K. V. et al. Chiral photonic super-crystals based on helical van der Waals homostructures. Preprint at 10.48550/arXiv.2309.16479 (2023).

[59] Ermolaev G (2022). Topological phase singularities in atomically thin high-refractive-index materials. Nat. Commun..

[60] Liu S (2016). Anisotropic coding metamaterials and their powerful manipulation of differently polarized terahertz waves. Light Sci. Appl..

[61] Sheldrick GM (2008). A short history of *SHELX*. Acta Crystallogr. Sect. A Found. Crystallogr..

[62] Sheldrick GM (2015). Crystal structure refinement with *SHELXL*. Acta Crystallogr. Sect. C Struct. Chem..

[63] Morimoto N (1954). The crystal structure of orpiment (As_2_S_3_) refined. Mineral. J..

[64] Mullen DJE, Nowacki W (1972). Refinement of the crystal structures of realgar, AsS and orpiment, As_2_S_3_*. Z. f.ür. Kristallographie.

[65] Krukau AV (2006). Influence of the exchange screening parameter on the performance of screened hybrid functionals. J. Chem. Phys..

[66] Kresse G, Hafner J (1993). Ab initio molecular dynamics for liquid metals. Phys. Rev. B.

[67] Frisenda R (2017). Micro-reflectance and transmittance spectroscopy: a versatile and powerful tool to characterize 2D materials. J. Phys. D: Appl. Phys..

