# Supplementary material for: Exploring van der Waals materials with high anisotropy: geometrical and optical approaches
Source: Light Sci Appl. 2024 Mar 8;13:68. doi: 10.1038/s41377-024-01407-3 (PMC10920635; doi:10.1038/s41377-024-01407-3)
Supplement: Supplementary file 1 — Supplementary Information [file 41377_2024_1407_MOESM1_ESM.docx]

**Supplementary Information for**

**Exploring van der Waals materials with high anisotropy: geometrical and optical approaches**

Aleksandr S. Slavich^1,†^,Georgy A. Ermolaev^2,†^, Mikhail K. Tatmyshevskiy^1^, Adilet N. Toksumakov^1^, Olga G. Matveeva^1^, Dmitriy V. Grudinin^2^, Arslan Mazitov^4^, Konstantin V. Kravtsov^1^, Alexander V. Syuy^2^, Dmitry M. Tsymbarenko^3^, Mikhail S. Mironov^2^, Sergey M. Novikov^1^, Ivan Kruglov^2^, Davit A. Ghazaryan^1,5^, Andrey A. Vyshnevyy^2^, Aleksey V. Arsenin^2,5^, Valentyn S. Volkov^2,5^, and Kostya S. Novoselov^6,7,8,*^

*^1^Moscow Center for Advanced Studies, Kulakova str. 20, Moscow, 123592, Russia*

*^2^Emerging Technologies Research Center, XPANCEO, Internet City, Emmay Tower, Dubai, United Arab Emirates*

*^3^Department of Chemistry, Lomonosov Moscow State University, Moscow, 119991, Russia*

*^4^Institute of Materials, École Polytechnique Fédérale de Lausanne, 1015 Lausanne, Switzerland*

*^5^Laboratory of Advanced Functional Materials, Yerevan State University, Yerevan 0025, Armenia*

*^6^National Graphene Institute (NGI), University of Manchester, Manchester, M13 9PL, UK*

*^7^Department of Materials Science and Engineering, National University of Singapore, Singapore, 03-09 EA, Singapore*

*^8^Institute for Functional Intelligent Materials, National University of Singapore, 117544, Singapore, Singapore*

^†^These authors contributed equally to this work

**Correspondence should be addressed to: kostya@nus.edu.sg*

**Table of Сontents**

**Supplementary Note 1: Characterization of As_2_S_3_ flakes**

**Supplementary Note 2: First-principle calculations**

**Supplementary Note 3: Polarization-dependent Raman spectroscopy**

**Supplementary Note 4: Processing of polarization-dependent transmittance spectra for As_2_S_3_**

**Supplementary Note 5: Mueller matrix ellipsometry analysis for determination of As_2_S_3_ optical constants**

**Supplementary Note 6: Scanning near-field optical microscopy of As_2_S_3_**

**Supplementary Note 7: Tabulated optical constants of As_2_S_3_**

**Supplementary Note 8: Transmittance calculations**

**Supplementary Note 9: Quarter-waveplate based on As_2_S_3_**

**Supplementary Note 1: Characterization of As_2_S_3_ flakes**

**Table S1.** Summary of the crystal structure and refinement details for As_2_S_3_.

| Formula | As_2_S_3_ |
| --- | --- |
| Formula weight | 246.02 |
| Diffractometer | Bruker D8 QUEST |
| Data collection method | *ω* scans |
| Temperature (K) | 297(2) |
| Crystal system | Monoclinic |
| Space group | *P 2_1_/n* |
| *a* (Å) | 4.2546(4) |
| *b* (Å) | 9.5775(10) |
| *c* (Å) | 11.4148(10) |
| *α* (°) | 90 |
| *β* (°) | 90.442(4) |
| *γ* (°) | 90 |
| *V* (Å^3^) | 465.12(8) |
| *Z* | 4 |
| Colour, habit | yellow, plate |
| Crystal dimensions (mm) | 0.169 × 0.151 × 0.015 |
| Density *D*_calc_ (g∙cm^-3^) | 3.513 |
| *μ* (mm^-1^) | 15.511 |
| Unique reflections (*R*_int_) | 1337 (0.0705) |
| Observed reflections [*I* > 2*σ*(*I*)] | 1018 |
| Parameters | 46 |
| *R*_1_[*I* > 2*σ*(*I*)], *ωR*_2_ | 0.0480, 0.1258 |
| Goodness of fit on *F*^2^ | 1.057 |
| Absorption correction | numerical |
| *T*_min_, *T*_max_ | 0.2170, 0.9763 |
| *ρ*_min_, *ρ*_max_ (eÅ^-3^) | -0.799, 2.353 |

**Table S2.** Interatomic distances (Å) and bond angles (°) in As_2_S_3_ structure. Symmetry codes: (i) 1+x, y, z; (ii) -0.5+x, 0.5 - y, 0.5+z; (v) -1+x, y, z; (vi) 0.5+x, 0.5-y, -0.5+z.

| Parameter | Distance | Parameter | Angle |
| --- | --- | --- | --- |
| As1−S1 | 2.2616(17) | S1−As1−S2^i^ | 99.24(6) |
| As1−S2^i^ | 2.2760(19) | S1−As1−S3 | 104.15(6) |
| As1−S3 | 2.2900(17) | S2^i^−As1−S3 | 94.50(7) |
| As2−S1 | 2.2484(18) | S1−As2−S2 | 98.83(6) |
| As2−S2 | 2.2844(17) | S1−As2−S3^ii^ | 92.67(6) |
| As2−S3^ii^ | 2.2926(17) | S2−As2−S3^ii^ | 104.98(6) |
|  |  | As2−S1−As1 | 103.62(7) |
|  |  | As1^v^−S2−As2 | 100.84(7) |
|  |  | As1−S3−As2^vi^ | 87.98(6) |

**Table S3.** Unit cell parameters and atomic position in crystal structure of As_2_S_3_.

|  | This work | Work^[1]^ | Work^[2]^ |
| --- | --- | --- | --- |
| *a* (Å) | 4.2546(4) | 4.22(5) | 4.256(2) |
| *b* (Å) | 9.5775(10) | 9.57(2) | 9.577(4) |
| *c* (Å) | 11.4148(10) | 11.46(4) | 11.475(5) |
| *β* (°) | 90.442(4) | 90.5(5) | 90.41(5) |
| As1 | x = 0.86181(16)  y = 0.19139(7)  z = 0.26514(5) | 0.857  0.190  0.267 | 0.86274(41)  0.19171(22)  0.26469(15) |
| As2 | x = 0.36056(16)  y = 0.32129(7)  z = 0.48652(5) | 0.357  0.323  0.484 | 0.36072(41)  0.32122(22)  0.48677(15) |
| S1 | x = 0.5099(4)  y = 0.12111(18)  z = 0.40041(13) | 0.500  0.120  0.395 | 0.50811(98)  0.12128(47)  0.40151(39) |
| S2 | x = 0.0115(4)  y = 0.39755(18)  z = 0.34757(13) | 0.987  0.397  0.355 | 0.01011(105)  0.39723(51)  0.34738(39) |
| S3 | x = 0.5603(4)  y = 0.29344(18)  z = 0.12185(13) | 0.590  0.293  0.125 | 0.55896(96)  0.29354(48)  0.12234(37) |
| *R*_1_ for all reflections | 0.0652 | 0.18 | 0.089 |


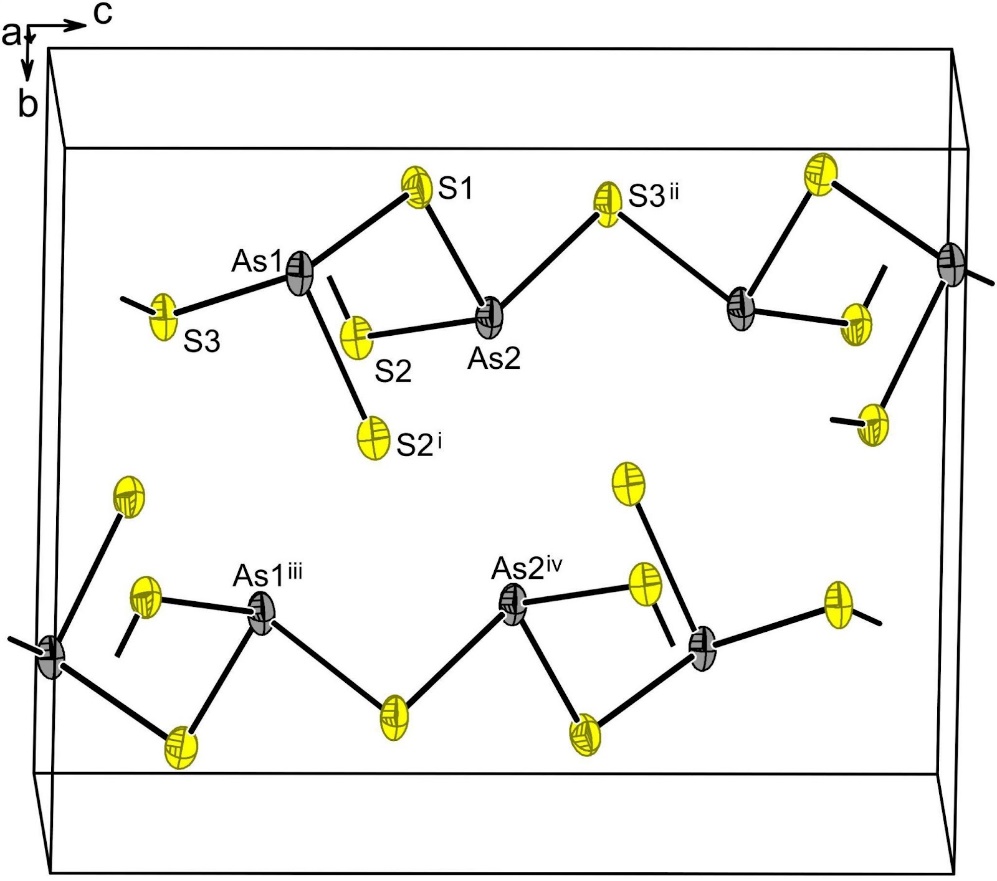


**Figure S1.** X-ray crystal structure of As_2_S_3_. Thermal ellipsoids correspond to 50% probability. Symmetry codes: (i) 1+x, y, z; (ii) -0.5+x, 0.5 - y, 0.5+z; (iii) 1.5-x, 0.5+y, 0.5-z; (iv) 1-x, 1-y, 1-z.

The monoclinic unit cell contains two symmetry related layered species of As2S3 stoichiometry located parallel to ac plane (Figure S1). Each species is formed by two crystallographically independent arsenic, As1 and As2, and three sulfur, S1-S3, atoms. Both As1 and As2 atom are covalently-bonded to three S atoms forming similar trigonal pyramides AsS_3_. The later are connected via bridging S-atoms and forms 2D layers. It worth noting that despite similar geometry six unique As-S distances are remarkably different (Table S2).

To verify the single-crystalline nature of As_2_S_3_ thin flakes we perform transmission electron microscopy (TEM) analysis. Figure S2a shows an optical image of an as-transferred As_2_S_3_ flake on SiN membrane. Figure S2b demonstrates the selected area electron diffraction (SAED) pattern of the As_2_S_3_ at the same flake. To confirm the As_2_S_3_ structure SAED pattern simulations were performed using the lattice parameters obtained from our X-ray diffraction result. The simulated SAED pattern for the [010] projection of As_2_S_3_ (Figure S2b) shows excellent agreement with experimental data. Energy-dispersive X-ray spectroscopy (EDX) measurements were further carried out to investigate the elemental composition of the specimen. The quantitative EDX analysis demonstrated a sufficient atomic stoichiometry As:S ≈ 38.1:61.9 as shown in Figure S2c. The obtained results confirmed the expected structure and high purity of the exfoliated As_2_S_3_ thin flakes.


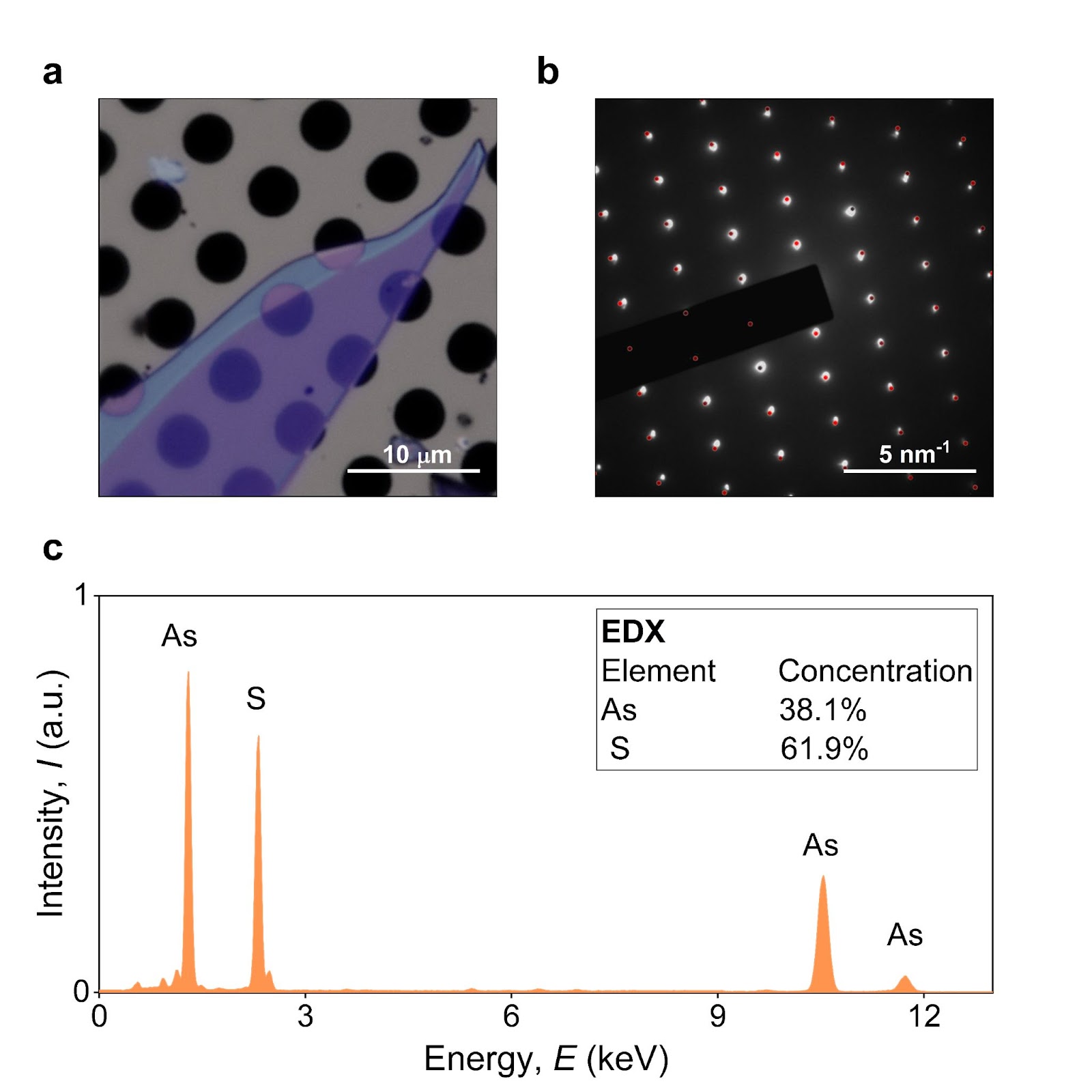


**Figure S2.** As_2_S_3_ TEM characterization. (a) Optical image of mechanically exfoliated As_2_S_3_ sheet transferred over holey SiN membrane. (b) Experimental selected area electron diffraction pattern across ac crystallographic plane. Colored dots indicate Miller index simulation pattern. (c) Energy-dispersive X-ray spectrum (EDX) of the transferred As_2_S_3_ flake.

**Supplementary Note 2: First-principle calculations**


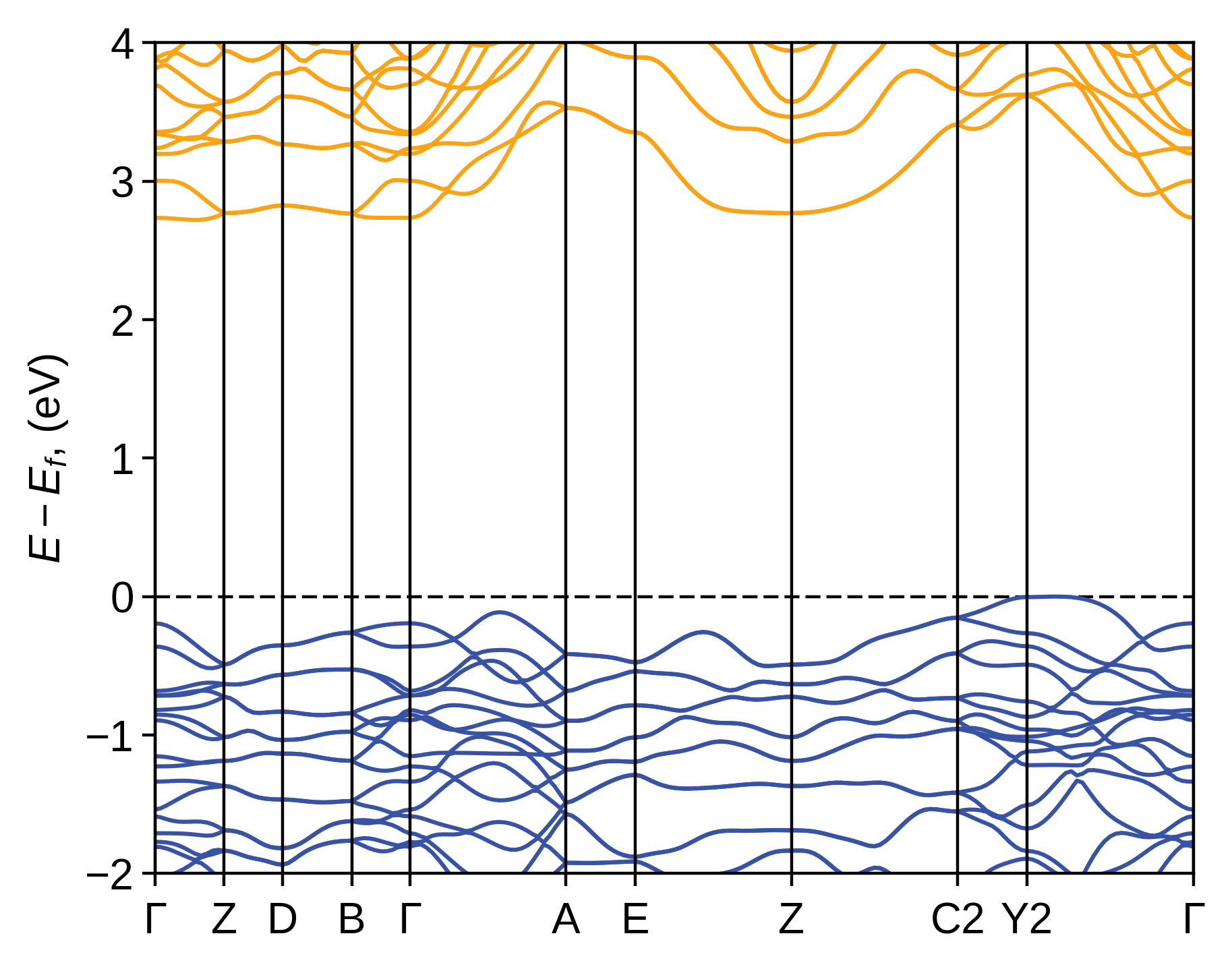


**Figure S3.** Bandstructure of As_2_S_3_ calculated within HSE06 hybrid functional using VASP code.

Optical properties of As_2_S_3_ were calculated within PBE^[3]^, HSE06^[4]^, PBE+GW^[5,6]^ and HSE06+GW^[7]^ approximations using the VASP code. In the GW approach, macro- and micro-dielectric tensors were also calculated. The comparison of different methods is shown in Figure S4. As expected, the best match between experiment and theory (Figure S4j) was for GW+HSE06 approach and micro-dielectric tensor for in-plane optical properties, and macro-dielectric tensor for out-of-plane optical properties.


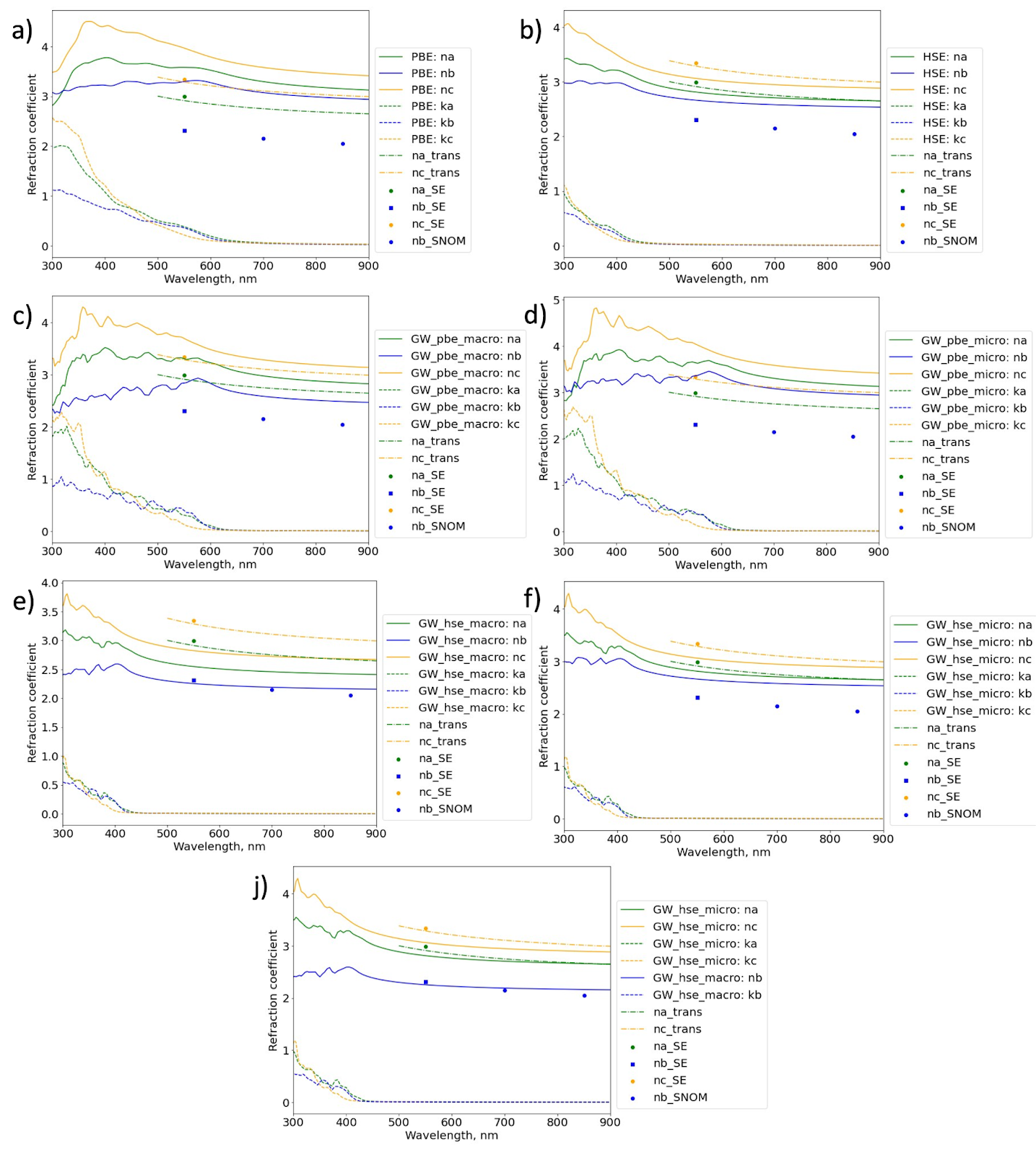


**Figure S4.** Optical properties of As_2_S_3_ calculated within a) PBE, b) HSE06, c) GW+PBE (from macro dielectric tensor), d) GW+PBE (from micro dielectric tensor), e) GW+HSE06 (from macro dielectric tensor), f) GW+HSE06 (from micro dielectric tensor), j) mixed micro-GW+HSE06 for in-plane and macro-GW+HSE06 for out-of-plane constants. Theoretically calculated data for refractive index is shown with solid line, experimental data - with dotted lines and points.

**Supplementary Note 3: Polarization-dependent Raman spectroscopy**

Lattice dynamics calculations of As_2_S_3_ were performed using the Phonopy^[8,9]^ and Phono3py^[10]^ codes, where VASP^[11]^ was used to calculate interatomic forces. Calculations were performed in the generalized form of gradient approximation (GGA), parametrized by the PBE exchange-correlation functional^[3]^ along with Grimme-D3 scheme^[12]^ to account for van der Waals interactions. For modeling ionic cores the PAW pseudopotentials^[13,14]^ were used treating the As 4s and 4p and the S 3s and 3p electrons as valence. A kinetic energy cutoff for the plane wave basis set was set to 350 eV. *k*-meshes of 12×5×4 and 4×3×4 subdivisions were used for the As_2_S_3_ primitive cell and supercells, respectively. The second-order force constants were calculated for 3×2×1 supercell (120 atoms) with a finite displacement step of 0.01 Å. A tolerance of 10^-8^ eV was set for total-energy calculations. We also considered a non-analytical term correction (NAC)^[15–17]^ for dynamical matrix to account for long-range dipole-dipole interactions.

Intensities of Raman peaks were calculated using the VASP package for both PBE and HSE06 functionals^[4]^. We used a default value of screening parameter = 0.2 for HSE06 with 25% mixing of exact exchange. For a static dielectric matrix and Born effective charges calculations a DFPT was used as implemented in VASP in the case of PBE. Finite electric field approach was used in the case of HSE06 with 0.002 eV Å^-1^ being the value of the applied electric field allowing one to avoid onset Zener tunneling in consistency with an evaluated band gap of 2.71 eV.

Linewidths were estimated using the Phono3py code. The third-order force constants were calculated for 3×2×1 supercell with atomic displacement of 0.03 Å. During post processing, the phonon lifetimes were sampled on regular Gamma-centered 21×21×21 *q*-point meshes.

A space group of As_2_S_3_ is P 2_1_/n (monoclinic crystal system), therefore Raman active modes are $A_{g}$ and $B_{g}$^[15-16]^. To compute polarized Raman spectra (Figure S5) we select only $A_{g}$ modes with consistency with the experiment: backscattering geometry (laser beam parallel to b-axis) and parallel configuration (polarizer parallel to analyzer). Figure S5 shows the calculated Raman spectra for incident laser-beam polarization along two crystalline axes and intensities of Raman-active modes as a function of polarization angle. Note, that the orientations of $A_{g}^{1}{-A}_{g}^{10}$ patterns are fixed relative to in-plane crystallographic directions. So the evaluated polar diagrams could be used to estimate directions of crystallographic axes in real experiment. As_2_S_3_ flakes used in this study were exfoliated onto a Schott glass and silicon substrates. Figures S6-7 show optical and atomic force microscopy (AFM) images of 60-nm-thick and 310-nm-thick As_2_S_3_ flakes. For the measurements of Raman spectra of the studied samples, Horiba LabRAM HR Evolution confocal scanning Raman microscope (Horiba Ltd., Kyoto, Japan) was used. All measurements were carried out using linearly polarized excitation of a 632.8 nm helium-neon laser. The studied samples were placed on a rotating table, which allowed us to measure angle-resolved Raman spectra. The Raman spectra were acquired at a range of angles from 0° to 360° with normal incidence angle by rotating a sample with a 5° step for 60-nm-thick As_2_S_3_ flake and with a 10° step for 310-nm-thick As_2_S_3_ flake. Figures S8-9 show the comparison of the polarized Raman modes intensities of the bulk As_2_S_3_ crystals with calculated data. Unfortunately, some peaks we cannot reliably resolve in terms of its polar diagram because of low peak intensity or small distance between peaks in experiment. However, it is clearly seen that calculated polar diagrams for the $A_{g}^{2}$, $A_{g}^{4}$-$A_{g}^{6}$ and $A_{g}^{9}$ modes are in excellent agreement with the experimental data. Therefore, these modes can be used to determine the crystalline axes of As_2_S_3_.


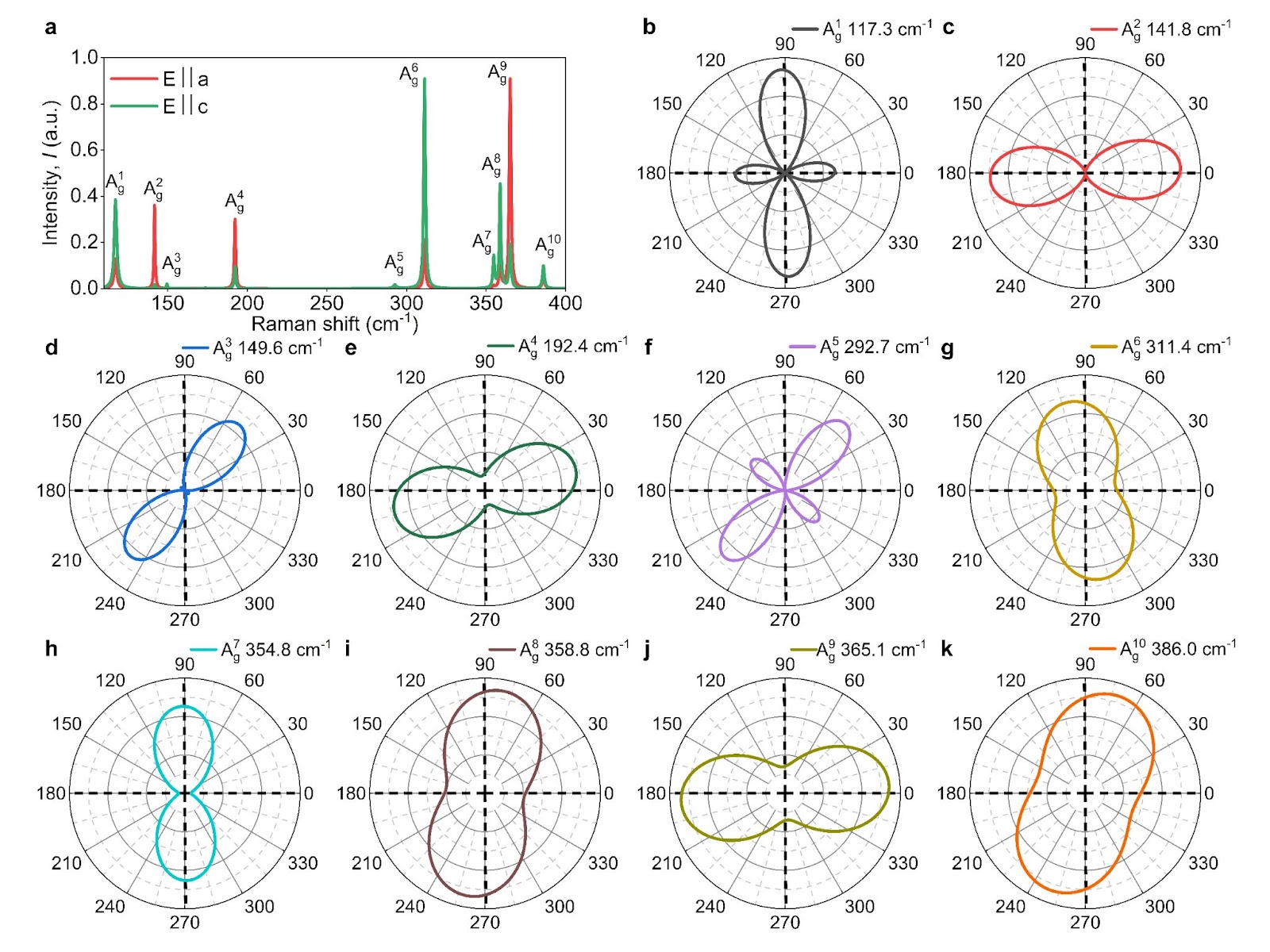


**Figure S5. Raman spectra of As_2_S_3_ from first-principle calculations.** (a) Calculated Raman spectra along *a*-axis and *c*-axis evaluated with polarizer being parallel to analyzer. (b-k) Polar diagrams for $A_{g}$ modes. Dashed lines indicate crystallographic axes directions: 0° corresponds to *a*-axis while 90.442° to *c*-axis.

**
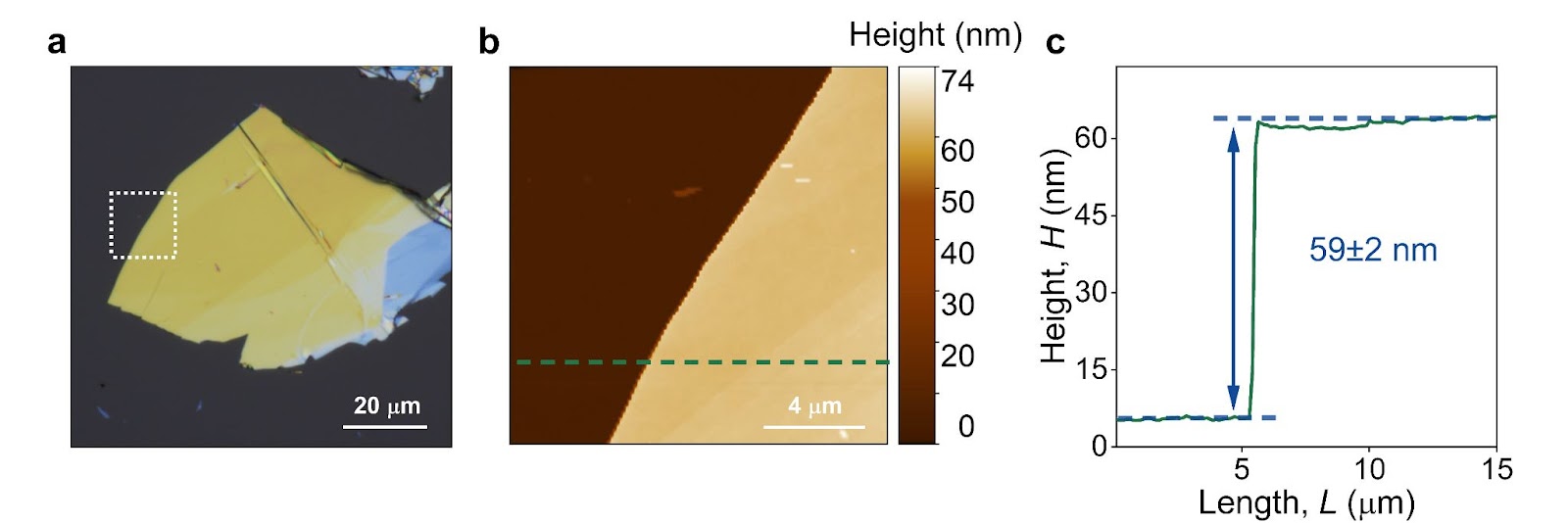
**

**Figure S6.** AFM characterization of the As_2_S_3_ flake (*t* ≈ 60 nm). (a) Optical image of the As_2_S_3_ flake on Schott glass. (b) AFM image outlined in (a). (c) Cross-section analysis along the green dashed line in (b).

**
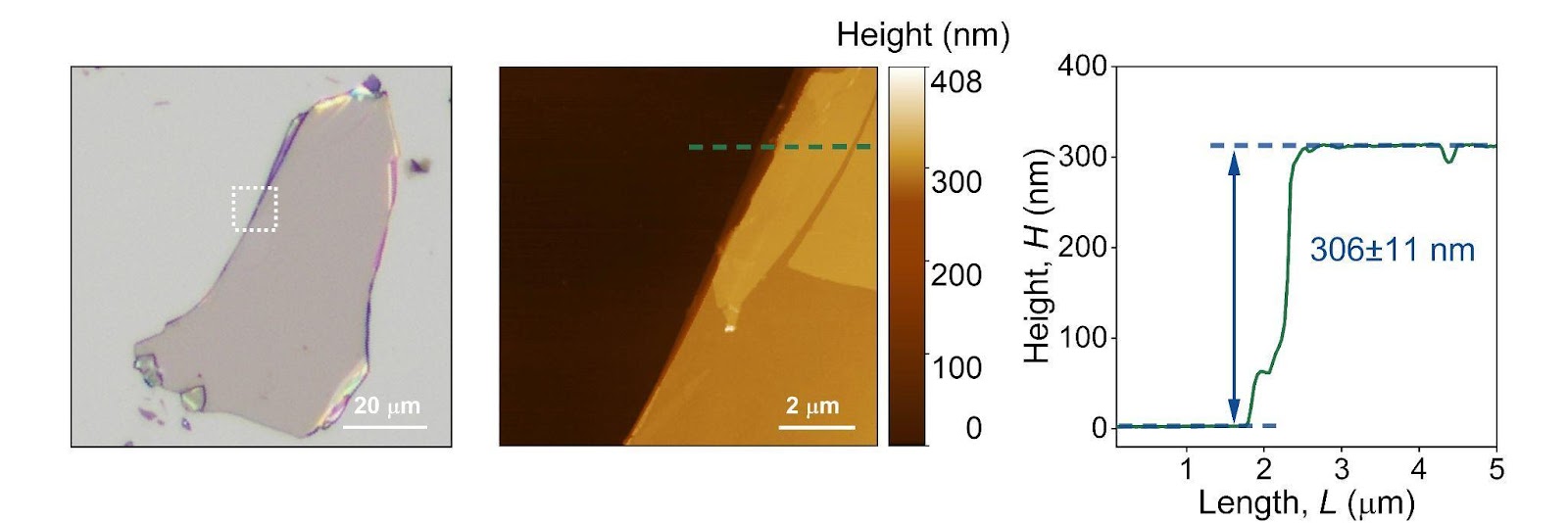
**

**Figure S7.** AFM analysis of the As_2_S_3_ flake exfoliated on silicon (*t* ≈ 310 nm). (a) Optical image of the As_2_S_3_ flake on Si. (b) AFM micrograph defined in (a). (c) Step height profile along the green dashed line in (b).


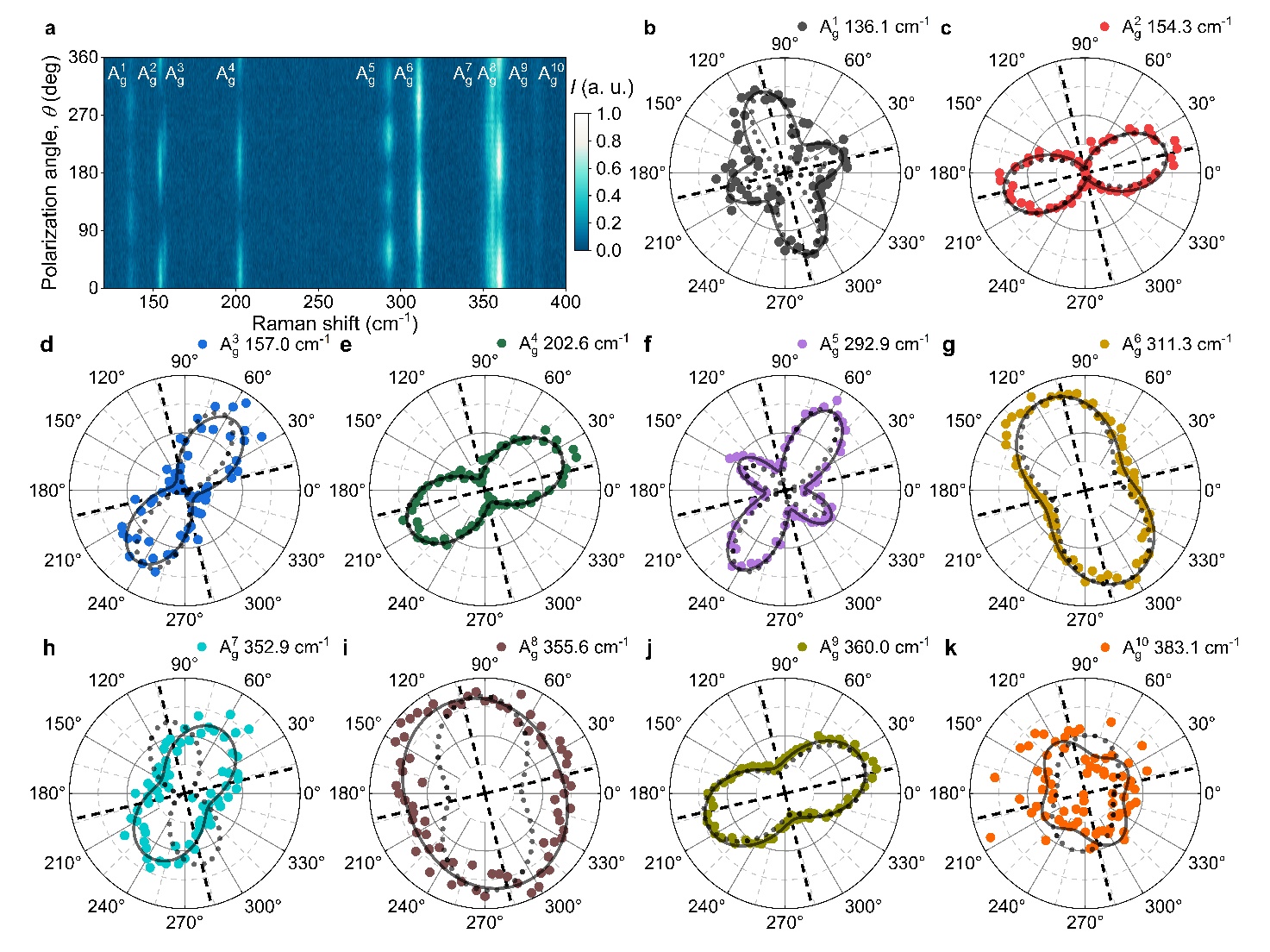


**Figure S8.** (a) Experimental Raman spectra of thin As_2_S_3_ (*t* ≈ 60nm) exfoliated on Schott glass. (b-k) Polar plots of the fitted peak intensities for ten Raman modes. Grey dotted curves correspond to Raman calculations. The data in polar diagrams are normalized to their maximum values, respectively. Dashed lines indicate *a* and *c* crystallographic directions.


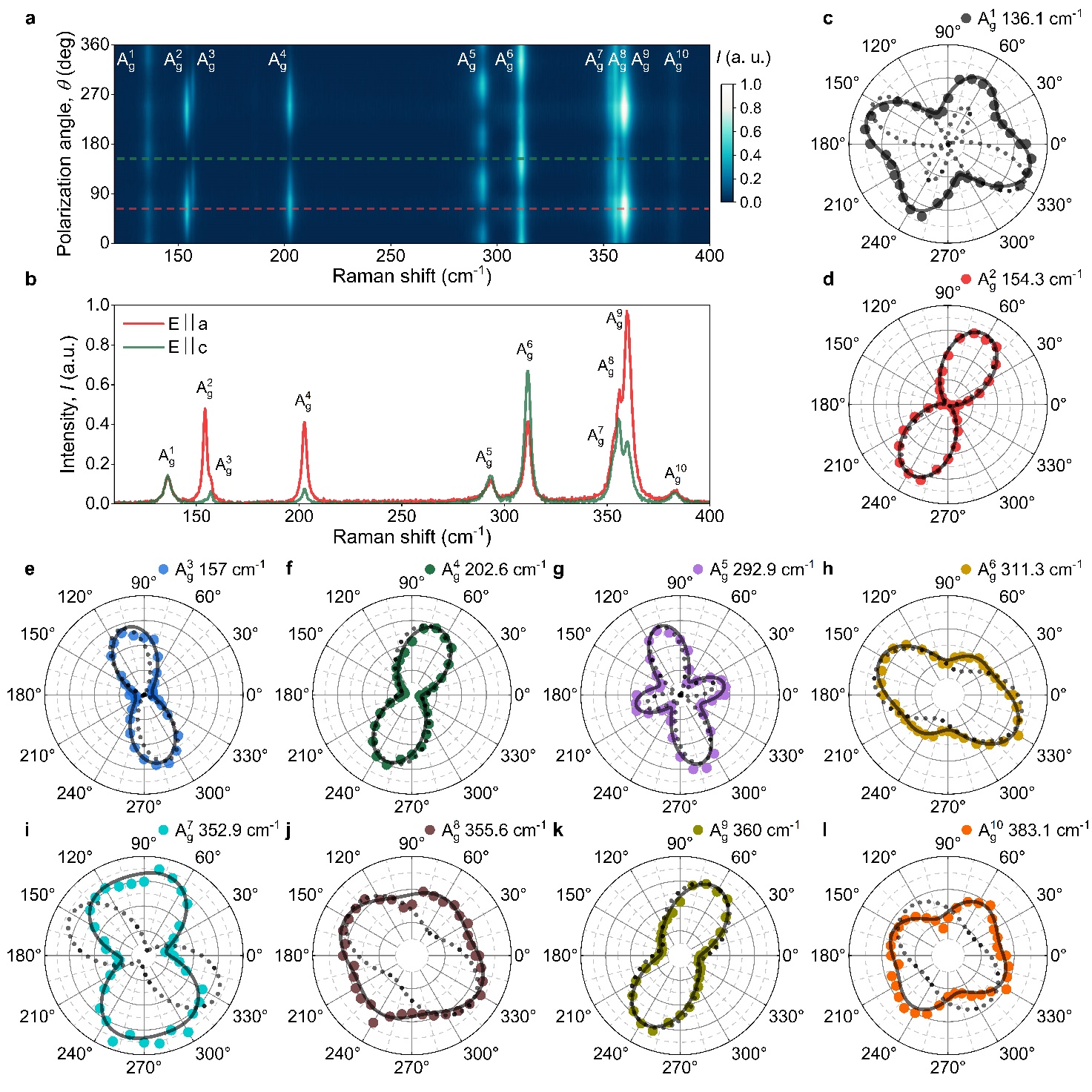


**Figure S9.** (a) Angle-resolved Raman intensity colormap of a bulk As_2_S_3_ flake (thickness, *t* ≈ 310 nm) acquired by rotating a sample in parallel-polarized configuration. Colored dashed lines indicate crystallographic directions. (b) Raman spectra along green and red lines on the map (a). (c-l) Polar plots of the normalized Raman intensity spectra for ten Raman modes. Dotted lines correspond to Raman calculations. The data in polar diagrams are normalized to their maximum values, respectively.

**Supplementary Note 4: Processing of polarization-dependent transmittance spectra for As_2_S_3_**

To determine optical axes position relative to the crystallographic axes, we performed micro-transmission measurements for the sample in parallel-polarized regime (see Methods). While the polarizer and the analyzer were fixed, we rotated the sample by 360° with a step of 5° and recorded the transmittance spectrum in the wavelength region of 500-850 nm for each angle. Figure S8 shows the transmittance color map which contains spectra for all rotation angles. Next, we linearly approximated the extracted experimental spectra over intervals *Δλ* of 1 nm and fitted them as follows:

$$T\left( \theta\right)=\left( E/E_{0} \right)^{2}=\left| a\left( cos \theta\right)^{2}+b\left( sin \theta\right)^{2}e^{i\Delta\varphi} \right|^{2}=$$

$$=a^{2}\left( cos \theta\right)^{4}+b^{2}\left( sin \theta\right)^{4}+2ab\left( cos \theta\right)^{2}\left( sin \theta\right)^{2}cos \Delta\varphi$$

where $T\left( \theta\right)$ are transmittance at a given wavelength $\lambda$ and polarization angle $\theta$, $\Delta\varphi$ is the phase retardance. We used the fitted curves to experimentally detect optical axes orientation.


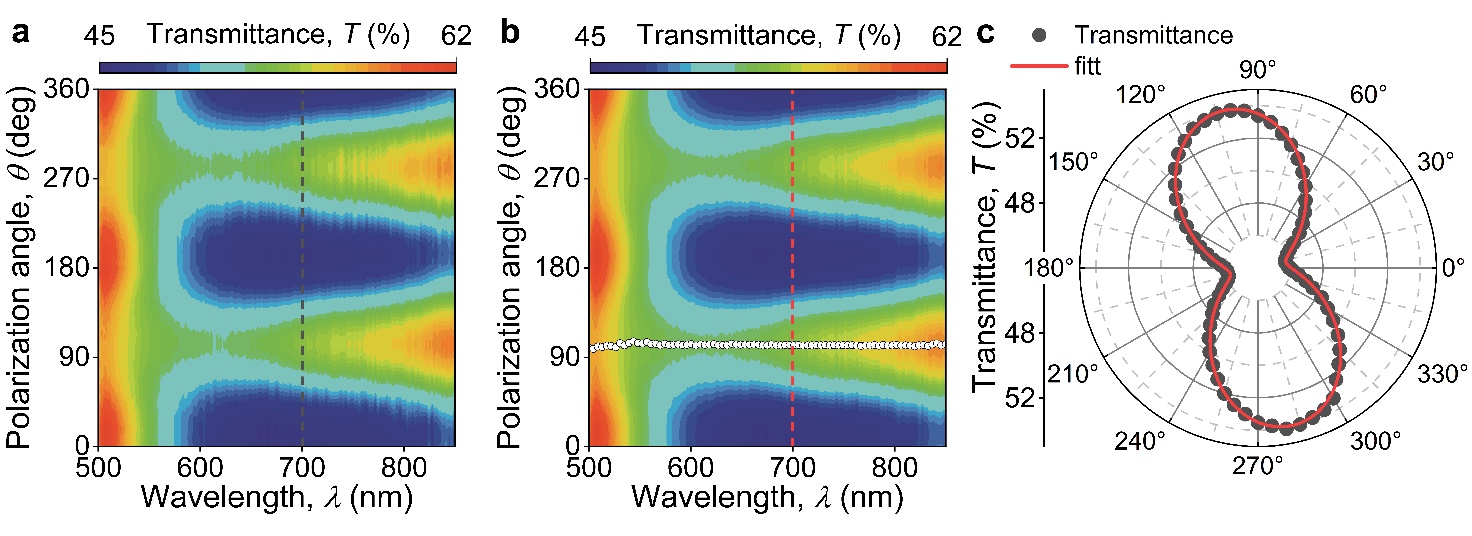


**Figure S10.** (a,b) Polarized micro-transmittance experimental and fitting maps of 60-nm-thick As_2_S_3_ flake. (c) Angle-dependent transmittance diagram is taken along dashed lines in the panels (a,b).

**Supplementary Note 5: Mueller matrix ellipsometry analysis for determination of As_2_S_3_ optical constants**

In order to obtain the full dielectric tensor of As_2_S_3_ we recorded Mueller matrix in 5° step sample rotation over 180° at 550 nm using Accurion nanofilm_ep4 ellipsometer. The resulted optical constants from the fitting of Mueller matrix (Figure S11) are plotted in Figure 3 of the main text.


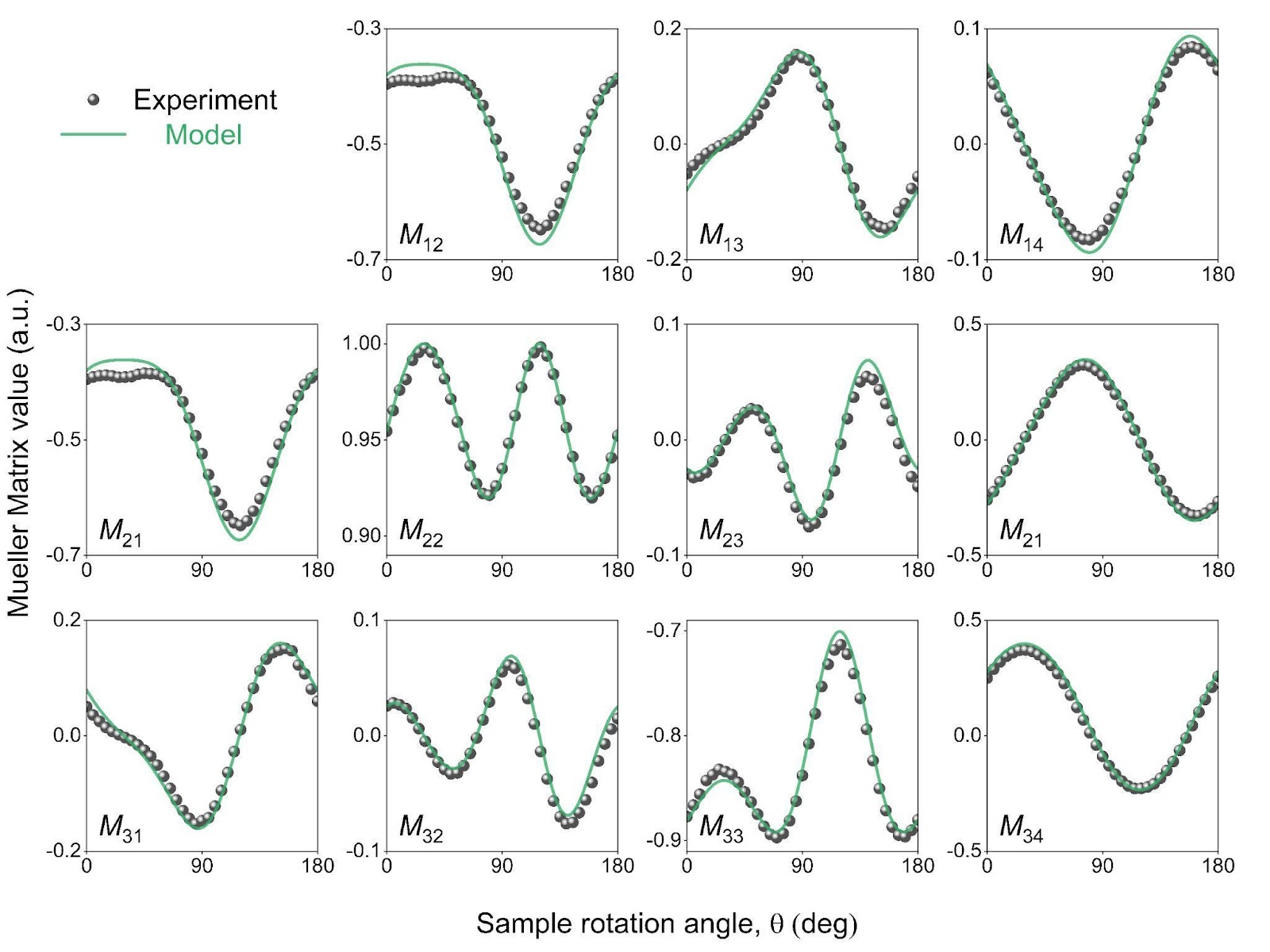


**Figure S11.** Mueller matrix for As_2_S_3_.

**Supplementary Note 6: Scanning near-field optical microscopy of As_2_S_3_**

For near-field measurements, we exfoliated the flake of the required thickness (*h* ≈ 250 nm) on Schott glass substrate. The thickness was determined so that only the fundamental TM mode could exist in the flake and there were no TM modes of higher orders. The measurements were carried out in the reflection mode. Meaning, the illumination of the sample and the collection of near-field information were performed by the same parabolic mirror. Since the material has an in-plane anisotropy of optical properties, we studied modes propagating in different directions as shown in Figures S12-13. It is well known that for the modes propagating in different directions, the near-field reflection measurements can exhibit a frequency shift that depends on the orientation of the edge that scatters or excites the mode, and can be described as^[18]^:

$$n_{\mathrm{eff}}=n_{\mathrm{obs}}+\cos\left( \beta\right)\sin\left( \alpha\right)$$

, where $n_{\mathrm{eff}}$ is the observable effective mode index, $n_{\mathrm{eff}}$ is the actual effective index of the mode, $\alpha$ is the angle between wavevector $k$ of the incident light and its projection of the samples surface $k_{\parallel}$, and $\beta$ is the angle between $k_{\parallel}$ and the direction of propagation of the mode in the sample. To determine the refractive index of the material along the *b* axis *n*_b_, we calculated the dispersion of planar TM_0_ modes at various refractive indices *n*_b_ and fixed *n*_a_ and *n*_c_ taken from the results of the polarized micro-transmittance (described in the main text).

**
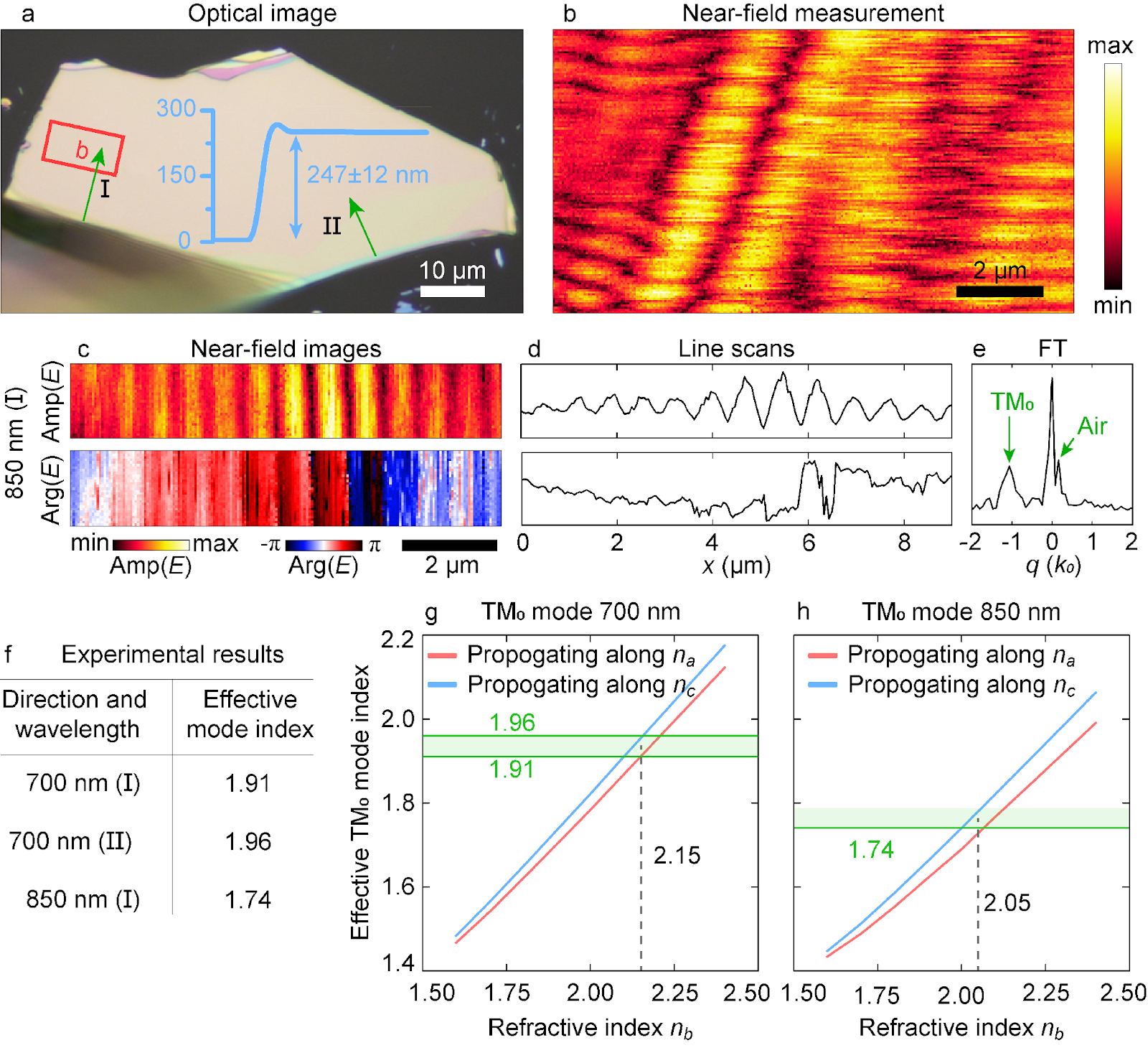
**

**Figure S12.** Near-field analysis of As_2_S_3_. (a) Optical image of the analyzed As_2_S_3_ flake. The inset shows AFM height profile. Green lines show propagation directions of studied planar mode. Red rectangle shows the area where measurement from b was taken. (b) Standard near-field image of the desired region. (c) Near-field image, amplitude Amp(*E*) and phase Arg(*E*), of the electric field *E* taken at 850 nm along direction I. (d) *x*-line scans taken from panel (c) and averaged over 1.8 μm. (e) Fourier transform (FT) amplitude of the complex near-field signal in (d). (f) Table with correspondence between the propagation direction, wavelength and effective mode index. (g) and (h) effective mode index of the fundamental TM mode as a function of the refractive index of As_2_S_3_ along *b*-axis for modes propagating along *a*-axis (red) and *c*-axis (blue).


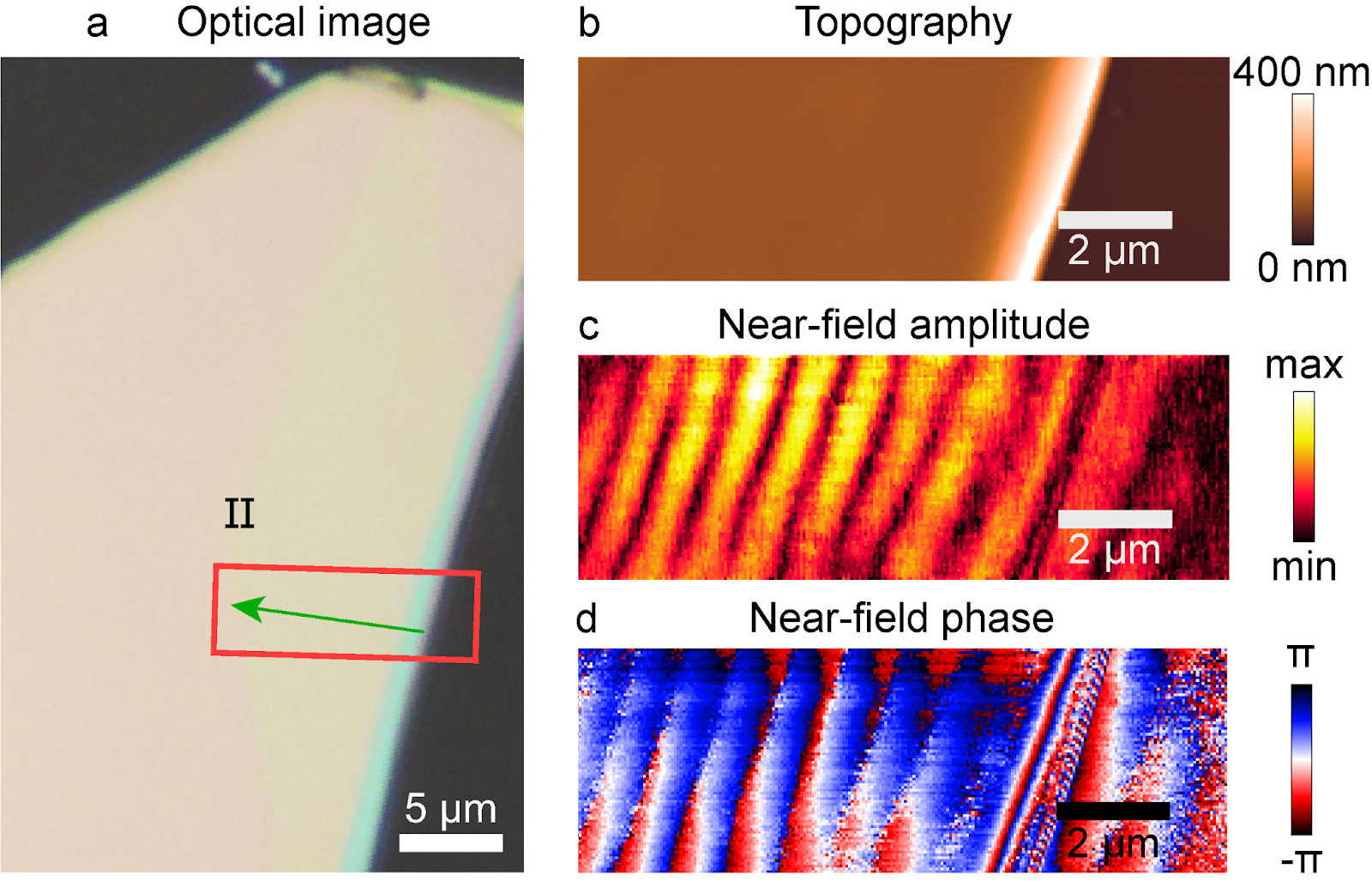


**Figure S13.** Near-field analysis of As_2_S_3_. (a) Optical image of the analyzed As_2_S_3_ flake. Green lines show propagation directions of studied planar mode. Red rectangle shows the area where measurement was taken. (b-d) Topography, near-field amplitude and near-field phase of the desired region (third harmonic).

**Supplementary Note 7: Tabulated optical constants of As_2_S_3_**

**Table S4.** Tabulated optical constants of As_2_S_3_.

| $\lambda$ (nm) | $n_{a}$ (in-plane) | $n_{b}$ (out-of-plane) | $n_{c}$ (in-plane) |
| --- | --- | --- | --- |
| 500 | 3.002 | 2.40425 | 3.38498 |
| 510 | 2.98205 | 2.38351 | 3.36294 |
| 520 | 2.96324 | 2.36395 | 3.34215 |
| 530 | 2.94548 | 2.34549 | 3.32253 |
| 540 | 2.9287 | 2.32804 | 3.30399 |
| 550 | 2.91283 | 2.31154 | 3.28646 |
| 560 | 2.8978 | 2.29591 | 3.26985 |
| 570 | 2.88355 | 2.2811 | 3.25411 |
| 580 | 2.87004 | 2.26704 | 3.23918 |
| 590 | 2.8572 | 2.2537 | 3.225 |
| 600 | 2.84501 | 2.24102 | 3.21152 |
| 610 | 2.8334 | 2.22895 | 3.1987 |
| 620 | 2.82236 | 2.21747 | 3.1865 |
| 630 | 2.81183 | 2.20653 | 3.17487 |
| 640 | 2.8018 | 2.19609 | 3.16378 |
| 650 | 2.79222 | 2.18614 | 3.15321 |
| 660 | 2.78308 | 2.17663 | 3.1431 |
| 670 | 2.77434 | 2.16755 | 3.13345 |
| 680 | 2.76599 | 2.15886 | 3.12422 |
| 690 | 2.758 | 2.15055 | 3.11539 |
| 700 | 2.75034 | 2.14259 | 3.10693 |
| 710 | 2.74301 | 2.13497 | 3.09883 |
| 720 | 2.73598 | 2.12766 | 3.09107 |
| 730 | 2.72924 | 2.12065 | 3.08362 |
| 740 | 2.72277 | 2.11392 | 3.07647 |
| 750 | 2.71656 | 2.10746 | 3.0696 |
| 760 | 2.71059 | 2.10126 | 3.06301 |
| 770 | 2.70485 | 2.09529 | 3.05667 |
| 780 | 2.69933 | 2.08955 | 3.05057 |
| 790 | 2.69402 | 2.08403 | 3.0447 |
| 800 | 2.6889 | 2.07871 | 3.03905 |
| 810 | 2.68398 | 2.07359 | 3.03361 |
| 820 | 2.67923 | 2.06866 | 3.02836 |
| 830 | 2.67466 | 2.0639 | 3.02331 |
| 840 | 2.67024 | 2.05931 | 3.01843 |
| 850 | 2.66599 | 2.05488 | 3.01373 |
| 860 | 2.66187 | 2.05061 | 3.00919 |
| 870 | 2.65791 | 2.04648 | 3.0048 |
| 880 | 2.65407 | 2.04249 | 3.00056 |
| 890 | 2.65036 | 2.03864 | 2.99647 |
| 900 | 2.64678 | 2.03491 | 2.99251 |

**Supplementary Note 8: Transmittance calculations**

In the experiment, incident light polarized by the first polarizer (P_1_) falls normally at the flake of As_2_S_3_ placed on the glass substrate (Figure S14). The transmitted light then passes through the measuring polarizer (analyzer) P_2_. We measure the dependence of the light intensity on the mutual orientation of the sample and polarizers P_1_ and P_2_. For the theoretical computations of the transmission coefficient, we solve Maxwell’s equations with appropriate boundary conditions.


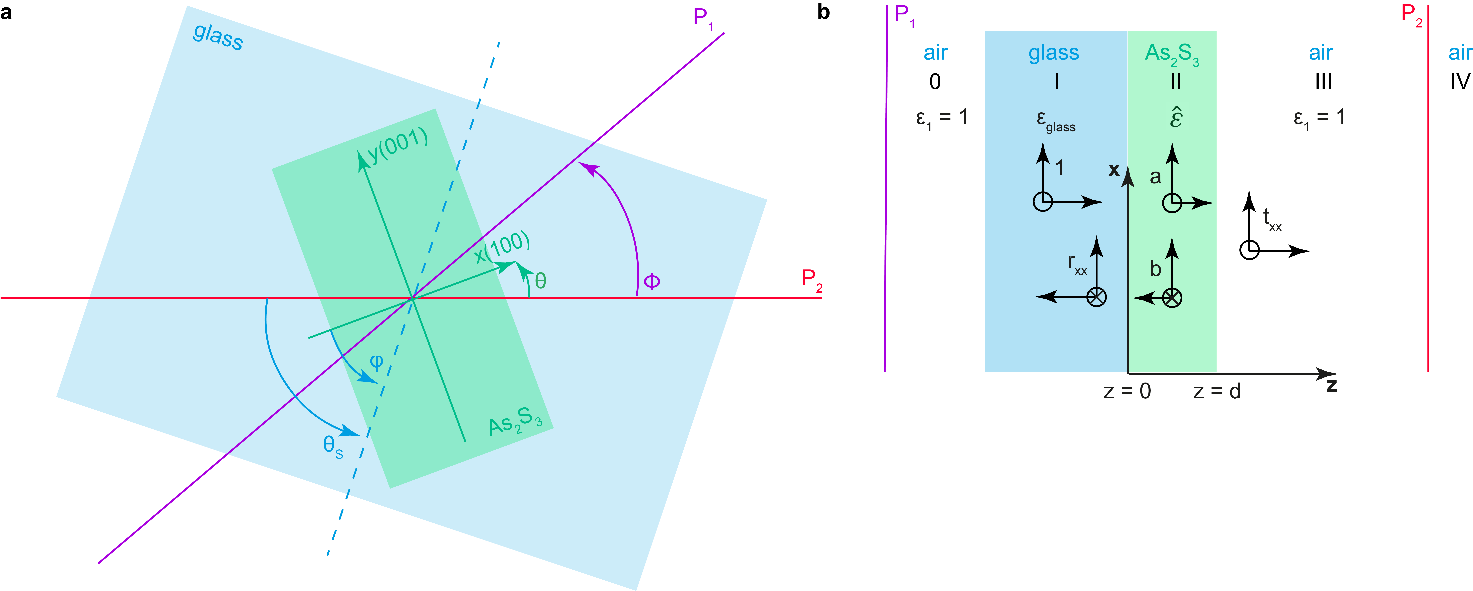


**Figure S14.** Schematic top- and side-view of the experimental setup. (a) Angle arrangement of the As_2_S_3_ plate, the substrate and polarizers P_1_ and P_2_. (b) Field distribution (in-plane electric components, *E*, signed as narrows and out-of-plane magnetic components, *H*, signed as circles with and without “*x*” depending on the direction).

First, we find the electric field in the regions 0 - IV (see Figure 13b). Neglecting the back-reflections in the glass plate, we can write:

$\boldsymbol{E}_{\mathrm{III}}=$ $\hat{T}\boldsymbol{E}_{I}\boldsymbol{=}$ $\hat{T}t_{air-glass}\boldsymbol{E}_{0}$ (8.1)

where $\hat{T}$ is the Jones matrix describing the transmission through the As_2_S_3_ slab. In the basis of the principal directions of As_2_S_3_ dielectric permittivity tensor, the Jones vector of an incident light can be written as:

$\mathbf{E}_{0}\boldsymbol{=}\left( \begin{matrix} \cos\left( \phi-\theta\right) \\ \sin\left( \phi-\theta\right) \end{matrix} \right)$ (8.2)

where $\theta$ is the angle between the *x*-axis of As_2_S_3_ and direction of the analyzer P_2_, and $\phi$ is the angle between P_1_ and P_2_.

Since matrix [
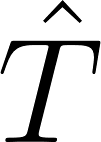
](https://www.codecogs.com/eqnedit.php?latex=%5Chat%7BT%7D#0) is diagonal, eq. (8.1) can be rewritten as:

$\boldsymbol{E}_{\mathrm{III}}\boldsymbol{=}$ $t_{air-glass}\left( \begin{matrix} t_{xx}\cos(\phi-\theta) \\ t_{yy}\sin\left( \phi-\theta\right) \end{matrix} \right)$ (8.3)

To find the components of the transmission matrix we treat an As_2_S_3_ slab as a Fabry–Pérot resonator

$t_{xx}=t_{I-II}^{x}\Phi t_{II-III}^{x}+t_{I-II}^{x}\Phi r_{II-III}^{x}\Phi r_{II-I}^{x}\Phi t_{II-III}^{x}+\ldots$ (8.4)

where $t_{i-j}=2n_{i}/(n_{i}+n_{j})$, and $r_{i-j}=t_{i-j}-1$ are the transmission and reflection amplitudes at the interface between regions *i* and *j* ($i,j=I,II,III)$ and $\Phi=\exp\left( ik_{x}d \right)$ is the phase accumulation factor which the wave with a wavenumber $k_{x}$ acquires upon propagation through the slab of a thickness *d*. The superscript *x* above reflection and transmission amplitudes signifies that for their calculation we assume $n_{II}=n_{x}$.

Summation of an infinite geometric progression results in

$t_{\mathrm{xx}}=\frac{t_{I-II}^{x}t_{II-III}^{x}\exp\left( ik_{x}d \right)}{1-r_{II-I}^{x}r_{II-III}^{x}\exp\left( 2ik_{x}d \right)}$ (8.5)

Similar equation with the trivial replacements is valid for $t_{yy}$.

The amplitude of the electric field of the detected wave after the analyzer P_2_ is found by projecting the Jones vector onto the analyzer direction

$E_{\mathrm{IV}}=t_{air-glass}\left( t_{xx}\cos(\phi-\theta)\cos\theta-t_{yy}\sin(\phi-\theta)\sin\theta\right)$ (8.6)

The transmission coefficient of our system reads:

$T= \frac{|{\mathbf{E}_{\mathrm{IV}}\boldsymbol{|}}^{2}}{|{\mathbf{E}_{0}\boldsymbol{|}}^{2}}={|E}_{\mathrm{IV}}\left. \right|^{2}$ (8.7)

A few notes should be added regarding the measurement procedure. Spectrophotometer measures the ratio of the transmission through our system to the transmission of the reference system which consists only of the glass slab. So, the number given by eq. (8.7) should be divided by ${|t_{air-glass}t_{glass-air}|}^{2}$:

$T_{\exp}= \frac{T}{{{|t}_{air-glass}t_{glass-air}|}^{2}}$ (8.8)

Furthermore, the orientation of the analyzer in our setup is fixed and unknown, therefore the experimental values of $\phi$ and $\theta$ differ from theoretical ones by a constant shift. However, by fitting the experimental data with eq. (8.8) we recover the orientation of the analyzer and, afterwards, the anisotropic refractive indices *n_x_* and *n_y_* of As_2_S_3_.

**Supplementary Note 9: Quarter-waveplate based on As_2_S_3_**

One of the main features of quarter-waveplates is their ability to transform a linearly polarized light into a circularly polarized. Assuming that |$t_{xx}$| = *A* and |$t_{yy}$| = *B*, we can rewrite the Jones vector after the As_2_S_3_ slab as:

$E_{III}=t_{air-glass}\left( A\cos\left( \varphi-\theta\right);Be^{i\delta}\sin\left( \varphi-\theta\right) \right)^{T}$ (8.9)

, where $\delta=Arg\left( t_{yy}/t_{xx} \right)$ and the common phase is discarded. This Jones vector corresponds to the circularly polarized light only if the phase retardance $\delta=\pm\pi/2$ and $\tan\left( \varphi-\theta\right)=\pm A/B$. The $\delta$ can be expressed as:

$\delta=\left( k_{x}-k_{y} \right)d+Arg\left( \frac{1-r_{II-I}^{y}r_{II-III}^{y}e^{2ik_{y}d}}{1-r_{II-I}^{x}r_{II-III}^{x}e^{2ik_{x}d}} \right)$ (8.10)

The first term here is the commonly used expression for the phase retardance in waveplates whereas the second term is related to the repeated reflections inside As_2_S_3_ slab hence can be named “Fabry–Pérot phase accumulation”.

For materials with weak in-plane optical anisotropy *n*_y_-*n*_x_, the second term is negligible and *A* = *B*. By contrast, a waveplate made from a material with a strong in-plane anisotropy exhibits noticeable linear dichroism (*A* $\neq$ *B*) and the Fabry–Pérot phase accumulation. These features must be properly addressed during the waveplate design and actual use. In particular, the waveplate can be thinner or thicker than predicted by the simple expression $\delta=\left( k_{x}-k_{y} \right)d$. Also, the polarization direction of an incident light should form an angle different from π/4 with the principal optical directions of the waveplate to be converted into circularly polarized light, i.e., $\varphi-\theta\neq$ π/4.


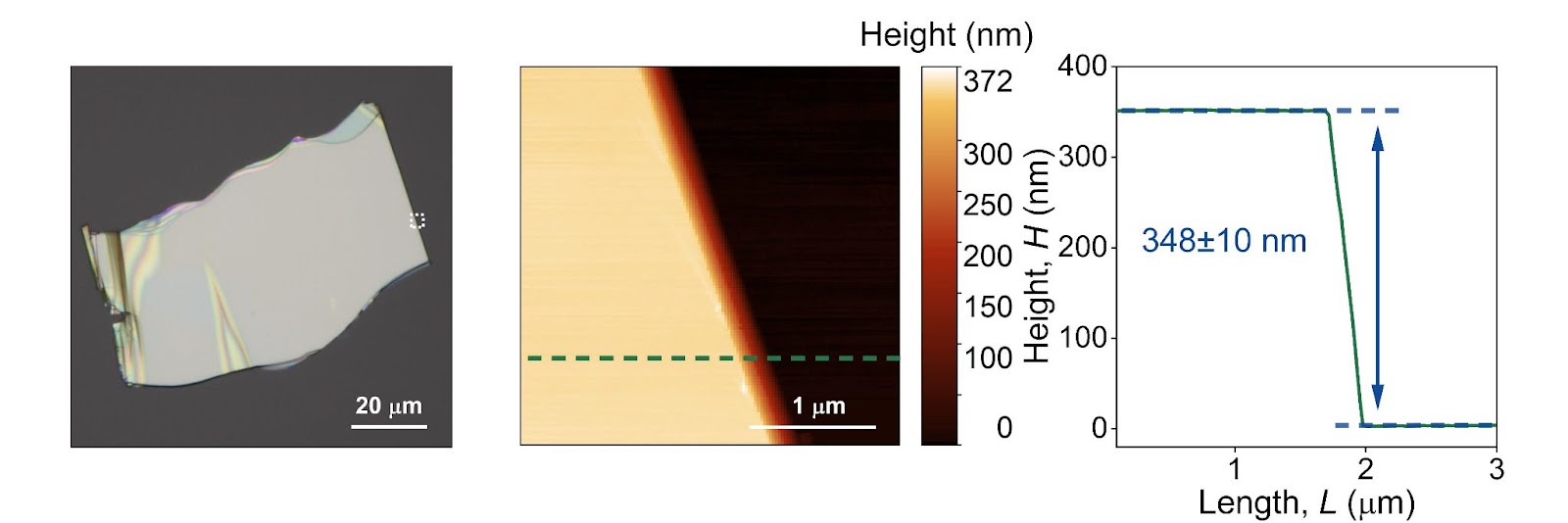


**Figure S15.** AFM characterization of the As_2_S_3_ flake quarter-waveplate (*t* = 345 nm from transmitted data fitting). (a) Optical image of the As_2_S_3_ flake on Schott glass. (b) AFM topographical scan over the region marked by dashed rectangle in panel (a). (c) Cross-sectional profile along the green dashed line.

**SUPPLEMENTARY REFERENCES**

[1] N. MORIMOTO, *Mineral. J.* **1954**, *1*, 160.

[2] D. J. E. Mullen, W. Nowacki, *Zeitschrift für Krist.* **1972**, *136*, 48.

[3] J. P. Perdew, K. Burke, M. Ernzerhof, *Phys. Rev. Lett.* **1996**, *77*, 3865.

[4] J. Heyd, G. E. Scuseria, M. Ernzerhof, *J. Chem. Phys.* **2003**, *118*, 8207.

[5] G. Kresse, J. Furthmüller, *Phys. Rev. B* **1996**, *54*, 11169.

[6] G. Kresse, J. Furthmüller, *Comput. Mater. Sci.* **1996**, *6*, 15.

[7] M. Shishkin, G. Kresse, *Phys. Rev. B - Condens. Matter Mater. Phys.* **2006**, *74*, 1.

[8] A. Togo, F. Oba, I. Tanaka, *Phys. Rev. B* **2008**, *78*, 134106.

[9] A. Togo, I. Tanaka, *Scr. Mater.* **2015**, *108*, 1.

[10] A. Togo, L. Chaput, I. Tanaka, *Phys. Rev. B* **2015**, *91*, 094306.

[11] G. Kresse, J. Hafner, *Phys. Rev. B* **1993**, *47*, 558.

[12] S. Grimme, S. Ehrlich, L. Goerigk, *J. Comput. Chem.* **2011**, *32*, 1456.

[13] P. E. Blöchl, *Phys. Rev. B* **1994**, *50*, 17953.

[14] G. Kresse, D. Joubert, *Phys. Rev. B* **1999**, *59*, 1758.

[15] X. Gonze, J.-C. Charlier, D. C. Allan, M. P. Teter, *Phys. Rev. B* **1994**, *50*, 13035.

[16] X. Gonze, C. Lee, *Phys. Rev. B* **1997**, *55*, 10355.

[17] R. M. Pick, M. H. Cohen, R. M. Martin, *Phys. Rev. B* **1970**, *1*, 910.

[18] D. Hu, X. Yang, C. Li, R. Liu, Z. Yao, H. Hu, S. N. G. Corder, J. Chen, Z. Sun, M. Liu, Q. Dai, *Nat. Commun.* **2017**, *8*, 1471.
